# The Central Role of cAMP in Regulating *Plasmodium falciparum* Merozoite Invasion of Human Erythrocytes

**DOI:** 10.1371/journal.ppat.1004520

**Published:** 2014-12-18

**Authors:** Amrita Dawn, Shailja Singh, Kunal R. More, Faiza Amber Siddiqui, Niseema Pachikara, Ghania Ramdani, Gordon Langsley, Chetan E. Chitnis

**Affiliations:** 1 Malaria Group, International Centre for Genetic Engineering and Biotechnology (ICGEB), New Delhi, India; 2 Malaria Parasite Biology and Vaccines Unit, Department of Parasites and Insect Vectors, Institut Pasteur, Paris, France; 3 Laboratoire de Biologie Cellulaire Comparative des Apicomplexes, Institut Cochin, INSERM U1016, CNRS UMR 8104, Paris, France; MRC National Institute for Medical Research, United Kingdom

## Abstract

All pathogenesis and death associated with *Plasmodium falciparum* malaria is due to parasite-infected erythrocytes. Invasion of erythrocytes by *P. falciparum* merozoites requires specific interactions between host receptors and parasite ligands that are localized in apical organelles called micronemes. Here, we identify cAMP as a key regulator that triggers the timely secretion of microneme proteins enabling receptor-engagement and invasion. We demonstrate that exposure of merozoites to a low K^+^ environment, typical of blood plasma, activates a bicarbonate-sensitive cytoplasmic adenylyl cyclase to raise cytosolic cAMP levels and activate protein kinase A, which regulates microneme secretion. We also show that cAMP regulates merozoite cytosolic Ca^2+^ levels via induction of an Epac pathway and demonstrate that increases in both cAMP and Ca^2+^ are essential to trigger microneme secretion. Our identification of the different elements in cAMP-dependent signaling pathways that regulate microneme secretion during invasion provides novel targets to inhibit blood stage parasite growth and prevent malaria.

## Author Summary

The blood stage of malaria parasites is responsible for all the morbidity and mortality associated with malaria. During the blood stage, malaria parasites invade and multiply within host erythrocytes. The process of erythrocyte invasion requires specific interactions between host receptors and parasite ligands. Many of the key parasite proteins that bind host receptors are localized in apical organelles called micronemes. Here, we demonstrate that cAMP serves as a key regulator that controls the timely secretion of microneme proteins during invasion. We show that exposure of merozoites to a low K^+^ environment, as found in blood plasma, leads to a rise in cytosolic cAMP levels due to activation of the cytoplasmic, bicarbonate-sensitive adenylyl cyclase β (PfACβ). A rise in cAMP activates protein kinase A (PKA), which regulates microneme secretion. In addition, cAMP triggers a rise in cytosolic Ca^2+^ levels through the Epac pathway. Increases in both cAMP and Ca^2+^ levels are essential for triggering microneme secretion. Identification of the different elements in the cAMP-dependent signaling pathways that regulate microneme secretion during invasion provides novel targets to block erythrocyte invasion, inhibit blood stage parasite growth and prevent malaria.

## Introduction

All the clinical symptoms of *Plasmodium falciparum* malaria are attributed to the blood stage of the parasite life cycle. The intra-erythrocytic stage of the life cycle is initiated when liberated *P. falciparum* merozoites invade and multiply within host red blood cells. Following the development of mature schizonts, next generation merozoites egress from infected erythrocytes and invade uninfected erythrocytes to start a new cycle of infection. Invasion of erythrocytes by *P. falciparum* merozoites is a complex multi-step process that is mediated by specific molecular interactions between red cell surface receptors and parasite protein ligands [1], [2]. A number of parasite ligands that mediate receptor binding during invasion reside in apical membrane-bound organelles known as micronemes and rhoptries [1], [2]. Timely secretion of these parasite ligands to the merozoite surface is critical for successful invasion [3], [4].

Microneme and rhoptry proteins are secreted from free *P. falciparum* merozoites in a two-step process [5]. First, exposure of extracellular merozoites to a low [K^+^] environment typical of blood plasma leads to a rise in cytosolic Ca^2+^ via a phospholipase C (PLC)-dependent pathway, which triggers translocation of microneme proteins such as 175 kD erythrocyte binding antigen (EBA175) and apical merozoite antigen-1 (PfAMA1) to the merozoite surface [5]. Subsequently, binding of EBA175 and its homologs to their erythrocyte receptors triggers secretion of rhoptry proteins such as PfRH2b, Clag3.1 and PfTRAMP [5], [6].

The pathways by which exposure of *P. falciparum* merozoites to a low K^+^ environment triggers a rise in cytosolic Ca^2+^ and microneme secretion are not understood. Here, we demonstrate that another ubiquitous second messenger, namely, 3'-5' cyclic adenosine monophosphate (cAMP), plays a central role in regulating cytosolic Ca^2+^ levels and microneme secretion during merozoite invasion of red blood cells. We demonstrate that exposure of merozoites to a low K^+^ environment as found in blood plasma activates the bicarbonate-sensitive cytoplasmic adenylyl cyclase β (PfACβ) leading to a rise in cytosolic cAMP levels and activation of protein kinase A (PKA), which regulates microneme secretion. In mammalian cells, the cAMP responsive PKA, which regulates diverse cellular processes in response to a rise in cytosolic cAMP levels, is composed of two catalytic subunits and two regulatory subunits [7]. Unlike mammalian cells, *P. falciparum* has a single inhibitory regulatory subunit (PfPKAr) and a single catalytic subunit (PfPKAc) [8]–[12]. As the PfPKAr subunit is not predicted to dimerize, the holoenzyme is likely to be composed of a one-to-one ratio of PfPKAr∶PfPKAc [12]. The PfPKAr subunit is predicted to have 2 cyclic nucleotide binding domains. When cAMP binds to one or both of these it provokes a conformational change that engenders the dissociation of the PfPKAr∶PfPKAc complex and activation of the released PfPKAc subunit that phosphorylates its specific substrates [8]–[12]. Like *Plasmodium*, *Toxoplasma* also encodes cAMP-dependent PKA and its inhibition leads to a block in tachyzoite growth [13]. Increase in cytosolic cAMP levels that would activate *Toxoplasma* PKA also mediates the tachyzoite to bradyzoite developmental switch [14]–[16]. In addition to activating PKA, we demonstrate that cAMP activates the Epac pathway [17] in *P. falciparum* merozoites, which triggers a rise in cytosolic Ca^2+^ leading to microneme release. cAMP thus plays a central role in regulating microneme secretion during red blood cell invasion by *P. falciparum* merozoites.

## Results

### cAMP production by adenylyl cyclase β (PfACβ) in *P. falciparum* merozoites

cAMP is produced in eukaryotic cells either by a transmembrane adenylyl cyclase (tmAC), which has a putative K^+^ channel at the N-terminus, or by a cytoplasmic soluble adenylyl cyclase (sAC). Homologs of both the tmAC (PF3D7_1404600) and sAC (PF3D7_0802600) (www.plasmodb.org) are encoded in *P. falciparum* genome [18], [19]. However, the gene encoding the *P. berghei* transmembrane AC (PbACα) has been knocked out with no deleterious effect on growth of blood stage parasites although sporozoites are impaired in hepatocyte infectivity [20]. Given that PbACα does not appear to have an essential role in blood stage growth we focused our attention on the soluble AC in *P. falciparum* (PfACβ).

**Figure 1:**
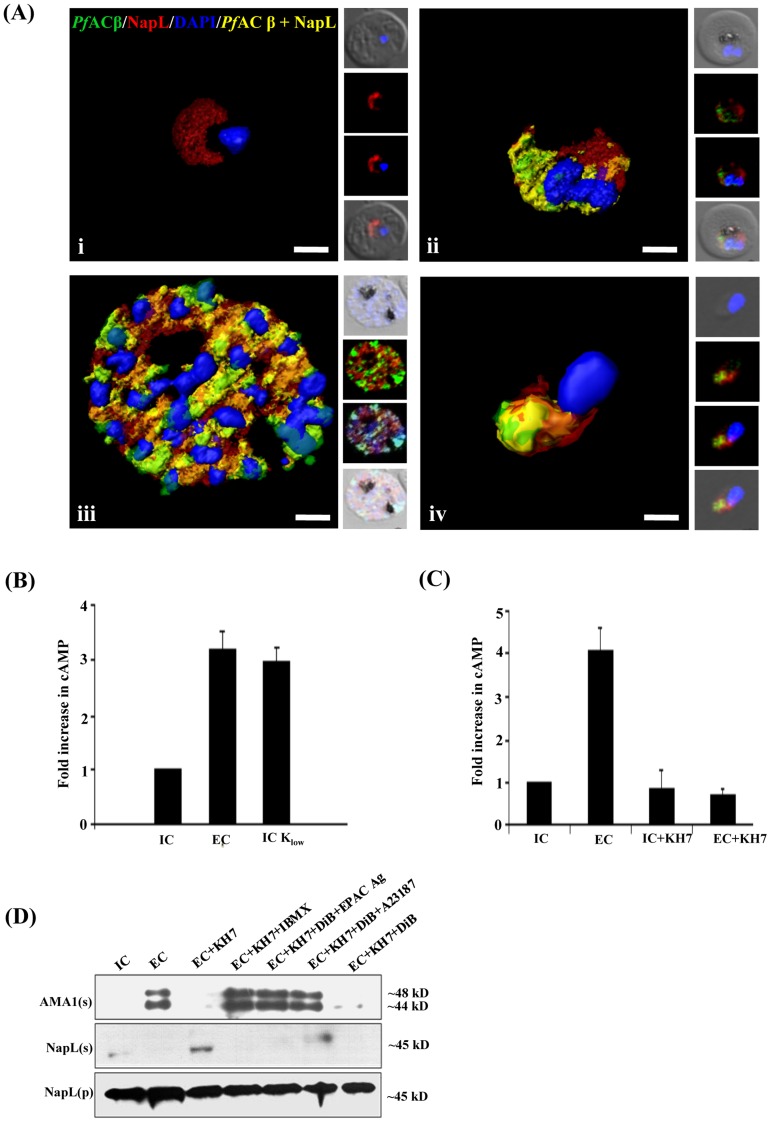
Production of cAMP in *P. falciparum* merozoites by adenylyl cyclase β (PfACβ) and microneme exocytosis. A) Expression of PfACβ in *P. falciparum* blood stages. Immunofluorescence assays (IFA) were used to detect PfACβ (green) in *P. falciparum* rings (i), trophozoites (ii), schizonts (iii) and merozoites (iv) using mouse antisera against a peptide derived from PfACβ. Nuclear DNA was stained with DAPI (blue). Rabbit antiserum against *P. falciparum* cytoplasmic protein PfNAPL (red) was used for co-localization. Yellow indicates overlap of PfACβ and PfNAPL. B) Exposure of *P. falciparum* merozoites to low K^+^ triggers production of cAMP. *P. falciparum* merozoites were isolated in buffer mimicking intracellular ionic environment (IC buffer – 140 mM KCl, 5 mM NaCl, 1 mM MgCl_2_, 5.6 mM glucose, 25 mM HEPES, pH 7.2) and transferred to buffer mimicking extracellular ionic environment (EC buffer – 5 mM KCl, 140 mM NaCl, 1 mM MgCl_2_, 2 mM EGTA, 5.6 mM glucose, 25 mM HEPES, pH 7.2) or IC-K_low_ buffer (IC-K_low_ buffer – 5 mM NaCl, 5 mM KCl, 135 mM choline-Cl, 1 mM EGTA, 5.6 mM glucose, 25 mM HEPES, pH 7.2). Levels of cytosolic cAMP in merozoite lysates were measured using a colorimetric cAMP Direct Immunoassay Kit (Calbiochem) as described in Materials & Methods. Total protein content in each merozoite sample was determined using Pierce Protein Assay Kit (Pierce). Amount of cAMP per µg of protein was determined for each merozoite sample and used to calculate fold increase compared to cAMP per µg of protein in control merozoites in IC buffer. Graphs represent mean fold change (± SD) in cAMP levels per µg of protein in merozoites under different conditions with respect to cAMP per µg of protein in merozoites in IC buffer from three independent experiments. C) Mammalian soluble AC inhibitor KH7 blocks rise in cytosolic cAMP levels following transfer of *P. falciparum* merozoites to low K^+^ buffer. *P. falciparum* merozoites were isolated in IC buffer and transferred to EC buffer with or without treatment with KH7. Levels of cAMP were measured in merozoite lysates as described above. Graphs represent mean fold change (± SD) in cAMP levels per µg of protein in merozoites under different conditions with respect to cAMP per µg of protein in merozoites in IC buffer from three independent experiments. Treatment with KH7 inhibits increase in cytosolic cAMP levels when merozoites are transferred from IC to EC buffer. D) Inhibition of microneme secretion by KH7. *P. falciparum* merozoites were transferred from IC to EC buffer with or without treatment with KH7. Secretion of PfAMA1 in merozoite supernatants (AMA(s)) was detected by Western blotting. Presence of cytoplasmic protein PfNapL was detected in *P. falciparum* merozoite supernatants (NapL(s)) and lysates of merozoite pellets (NapL(p)) under different conditions by Western blotting to control for merozoite lysis and number of merozoites used in the different conditions, respectively. Treatment of merozoites with KH7 inhibits secretion of microneme protein PfAMA1 following transfer from IC to EC buffer. Treatment of merozoites with KH7+IBMX, KH7+DiB+Epac Agonist and KH7+DiB+A23187 restores microneme secretion. However, treatment with KH7+DiB does not restore microneme secretion.

Mouse antiserum raised against a synthetic peptide derived from PfACβ detects a protein of expected size (>250 kD) in schizont and merozoite lysates by Western blotting (S1 Figure). Detection of PfACβ by immunofluorescence assay (IFA) demonstrates that it co-localizes in late stage schizonts and merozoites with the cytoplasmic protein PfNapL [21] (Fig. 1). PfACβ is thus expressed in *P. falciparum* merozoites and is localized in the cytoplasm.

Exposure of *P. falciparum* merozoites to an ionic environment with low K^+^ as found in blood plasma triggers a rise in cytosolic Ca^2+^ and secretion of microneme proteins [5]. To determine if exposure of free merozoites to a low K^+^ environment also leads to a rise in cytosolic cAMP, we measured cAMP levels in purified merozoites suspended in a buffer that mimics the intracellular ionic environment (IC buffer – 140 mM KCl, 5 mM NaCl, 1 mM MgCl_2_, 5.6 mM glucose, 25 mM HEPES, pH 7.2) and following transfer to a buffer that mimics extracellular ionic environment with low K^+^ levels (EC buffer – 5 mM KCl, 140 mM NaCl, 1 mM MgCl_2_, 2 mM EGTA, 5.6 mM glucose, 25 mM HEPES, pH 7.2). cAMP levels were also measured before and after merozoites were transferred from IC to IC-K_low_ buffer (IC-K_low_ buffer – 5 mM NaCl, 5 mM KCl, 135 mM choline-Cl, 1 mM EGTA, 5.6 mM glucose, 25 mM HEPES, pH 7.2) to determine if change in environmental K^+^ level alone can trigger changes in cAMP levels. There is a distinct rise in cAMP when merozoites are transferred from IC to EC buffer, or from IC to IC-K_low_ buffer (Fig. 1B). Importantly, transfer of free merozoites to a low K^+^ buffer in the presence of KH7, which inhibits mammalian sAC [19], blocks the rise in cytosolic cAMP (Fig. 1C).

### Regulation of microneme secretion by cAMP

We monitored the secretion of microneme protein PfAMA1 following the transfer of free *P. falciparum* merozoites from IC to EC buffer with or without prior treatment with KH7. PfAMA1 is proteolytically cleaved into 48 kD and 44 kD fragments that are released into the supernatant following translocation to the merozoite surface [22]. PfAMA1 was detected in merozoite supernatants following transfer from IC to EC buffer by Western blotting using anti-PfAMA1 sera (Fig. 1D). Transfer of extracellular merozoites from IC to EC buffer in the presence of KH7 blocks the secretion of PfAMA1 (Fig. 1D). Treatment of merozoites with KH7 in the presence of 3-isobutyl-1-methylxanthine (IBMX), which inhibits 3’,5’-cAMP phosphodiesterase (PDE) and maintains high cAMP levels in cells [23], reverses the block in microneme secretion (Fig. 1D). Secretion of another microneme protein, EBA175, is also inhibited if merozoites are treated with KH7 prior to transfer from IC to EC buffer (S2 Figure).

### cAMP production in *P. falciparum* merozoites upon exposure to a low K^+^ environment

**Figure 2:**
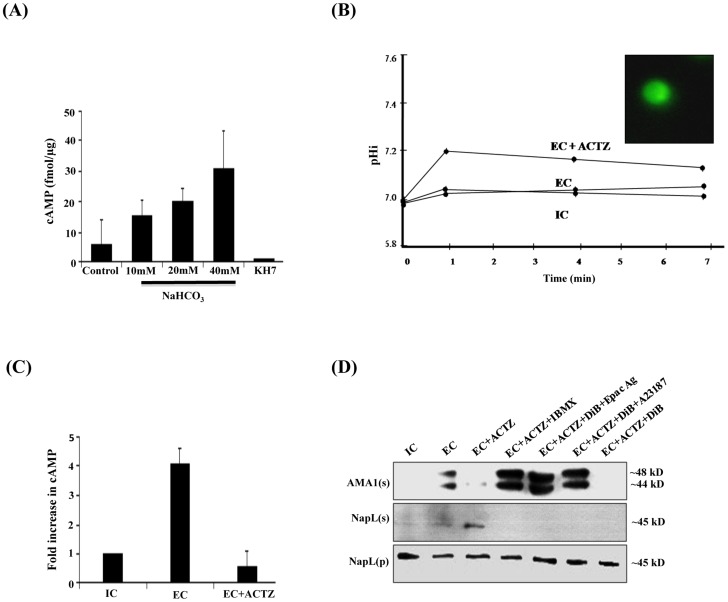
Production of cAMP by HCO_3_^−^ sensitive PfACβ and regulation of microneme secretion. A) HCO^−^_3_ sensitive adenylyl cyclase activity in *P. falciparum* merozoite lysates. *P. falciparum* merozoite lysates were incubated with increasing concentrations of NaHCO_3_ (10 mM, 20 mM and 40 mM) and ATP for 30 min at 30°C and production of cAMP was measured. Levels of cAMP are reported (mean + SD from three independent experiments) as femtomoles per µg of merozoite protein. Merozoite lysates without exogenous ATP were used as negative controls. Production of cAMP increased with increasing concentrations of NaHCO_3_. Addition of KH7 in presence of 40 mM NaHCO_3_ inhibited cAMP production. B) Intracellular pH (pH_i_) of *P. falciparum* merozoites in different ionic environments with and without treatment with carbonic anhydrase (CA) inhibitor acetazolamide (ACTZ). *P. falciparum* merozoites loaded with the pH-sensitive fluorescent dye BCECF-AM (inset) were transferred from IC to EC buffer with or without prior treatment with ACTZ. Fluorescence signal from BCECF was measured and used to determine pH_i_ using a standard curve (S6 Figure) as described in Materials and Methods. The pH_i_ of *P. falciparum* merozoites remains unchanged following transfer from IC to EC buffer. Pre-treatment of *P. falciparum* merozoites with ACTZ results in a rise in pH_i_ following transfer from IC to EC buffer (EC+ACTZ). C) ACTZ blocks rise in intracellular cAMP following transfer of *P. falciparum* merozoites from IC to EC buffer. *P. falciparum* merozoites were transferred from IC to EC buffer with or without treatment with ACTZ. Fold change (mean ± SD) in cAMP levels is reported from 3 independent experiments. Pre-treatment with ACTZ inhibits rise in cAMP in merozoites following transfer from IC to EC buffer. D) ACTZ blocks microneme secretion following transfer of *P. falciparum* merozoites from IC to EC buffer. *P. falciparum* merozoites were transferred form IC to EC buffer with or without prior treatment with CA inhibitor, ACTZ. Secretion of PfAMA1 in merozoite supernatants (AMA1(s)) was detected by Western blotting. Presence of cytoplasmic protein PfNapL was detected in *P. falciparum* merozoite supernatants (NapL(s)) and lysates of merozoite pellets (NapL(p)) under different conditions by Western blotting to control for merozoite lysis and number of merozoites used in the different conditions, respectively. Treatment of merozoites with ACTZ prior to transfer from IC to EC buffer inhibits secretion of microneme protein PfAMA1. Treatment of merozoites with ACTZ+IBMX, ACTZ+DiB+Epac Agonist and ACTZ+DiB+A23187 restores microneme secretion. However, treatment with ACTZ+DiB does not restore microneme secretion.

Production of cAMP by soluble adenylyl cyclases is regulated by HCO_3_^−^ ions in diverse organisms [24]–[26]. Incubation of *P. falciparum* merozoite lysates with ATP and different concentrations of HCO_3_^−^ ions showed that increasing amounts of cAMP are produced in presence of increasing concentrations of HCO_3_^−^ ions suggesting the presence of a HCO_3_^−^ sensitive adenylyl cyclase activity (Fig. 2A). Moreover, cAMP production is inhibited by KH7 (Fig. 2A). These observations confirm the presence of a HCO_3_^−^-sensitive ACβ in free *P. falciparum* merozoites.

Cytosolic pH (pH_i_) is controlled in eukaryotic cells by carbonic anhydrase (CA), which catalyzes the reversible hydration of CO_2_ to produce H^+^ and HCO_3_^−^ (CO_2_+H_2_O↔HCO_3_^−^+H^+^) [27]. Acetazolamide (ACTZ), a specific inhibitor of CA, can disrupt intracellular pH homeostasis [28], [29]. We tested whether the transfer of free merozoites from IC to EC buffer in presence of ACTZ affects pH_i_. *P. falciparum* merozoites collected in IC buffer were loaded with the membrane permeable pH-sensitive fluorescent dye BCECF-AM [30] and transferred to EC buffer in the presence or absence of ACTZ. pH_i_ does not change when free merozoites are transferred from IC to EC buffer. In contrast, there is a rapid rise in pH_i_ when free merozoites are transferred from IC to EC buffer in presence of ACTZ (Fig. 2B). These observations indicate that PfCA activity is necessary for maintenance of pH_i_ when merozoites are transferred to a low K^+^ environment.

The observation that pH_i_ rises when merozoites are transferred to EC in presence of ACTZ is consistent with a model in which PfCA maintains pH_i_ by producing H^+^ and HCO_3_^−^. To test whether the generation of HCO_3_^−^ in free merozoites following transfer from IC to EC buffer leads to activation of PfACβ, we measured levels of cAMP in merozoites with or without treatment with ACTZ. Treatment of merozoites with ACTZ prior to transfer from IC to EC buffer inhibits rise in cAMP (Fig. 2C) as well as secretion of PfAMA1 (Fig. 2D). Treatment of *P. falciparum* merozoites with ACTZ in presence of IBMX reverses the block in microneme secretion (Fig. 2D). These results suggest that when free merozoites are exposed to an extracellular-like low K^+^ environment, the production of HCO_3_^−^ by PfCA to maintain pH_i_ leads to activation of PfACβ and generation of cAMP, which triggers microneme secretion. Exposure of merozoites in IC buffer to increasing concentrations of HCO_3_^−^ also leads to a dose-dependent increase in microneme secretion (S3 Figure) confirming that HCO_3_^−^ plays a key role in regulating microneme secretion.

### Potential role of cAMP-responsive protein kinase A (PKA) in regulation of microneme secretion

**Figure 3:**
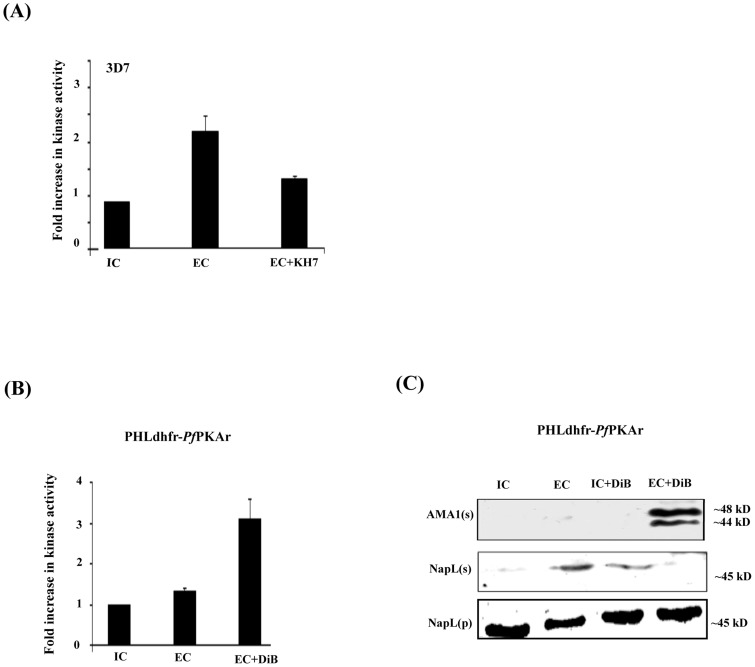
cAMP responsive kinase activity in *P. falciparum* merozoites under different ionic conditions and microneme secretion. A) Kemptide phosphorylation by *P. falciparum* 3D7 merozoite lysates under different ionic environments. *P. falciparum* 3D7 merozoites were transferred from IC buffer to EC buffer with or without treatment with ACβ inhibitor, KH7. Merozoite lysates made under different conditions were incubated with kemptide, a protein kinase A (PKA) substrate, and γ-^32^P-ATP. Fold change (mean ± SD) in phosphorylation of kemptide under different ionic conditions is reported relative to phosphorylation in IC buffer from 3 independent experiments. B) Kemptide phosphorylation by lysates of transgenic *P. falciparum* PHL dhfr-*Pf*PKAr merozoites under different ionic environments. *P. falciparum* PHL dhfr-*Pf*PKAr merozoites were isolated in IC buffer and transferred to EC buffer with or without treatment with dibutryl-cAMP (DiB). Phosphorylation of kemptide by merozoite lysates made under different conditions was measured in presence of γ-^32^P-ATP. Fold change in kemptide phosphorylation (mean ± SD) by merozoite lysates under different conditions is reported relative to that in IC buffer from 3 independent experiments. C) Microneme secretion in *P. falciparum* PHL dhfr-PfPKAr merozoites. *P. falciparum* PHL dhfr-PfPKAr merozoites were transferred from IC to EC buffer, IC buffer+DiB, or EC buffer+DiB and secretion of PfAMA1 into merozoite supernatants (AMA1(s)) was detected by Western blotting. Presence of cytoplasmic protein PfNapL was detected in *P. falciparum* merozoite supernatants (NapL(s)) and lysates of merozoite pellets (NapL(p)) under different conditions by Western blotting to control for merozoite lysis and number of merozoites used in the different conditions, respectively. PfAMA1 was not secreted when *P. falciparum* PHL dhfr-PfPKAr merozoites were transferred from IC to IC+DiB, or from IC to EC buffer. PfAMA1 was secreted when *P. falciparum* PHL dhfr-PfPKAr merozoites were transferred from IC to EC+DiB.

PKA commonly plays a role as a cAMP-responsive effector in signaling pathways [7]. To investigate the potential role of PfPKA in microneme discharge, we measured phosphorylation of kemptide, a specific substrate of PKA, by lysates of *P. falciparum* merozoites in IC and EC buffers with and without addition of KH7. There is a distinct increase in kemptide phosphorylation with lysates made from merozoites in EC buffer compared to IC buffer (Fig. 3A). Pre-treatment with KH7 inhibits this increase in kemptide phosphorylation by merozoite lysates in EC buffer suggesting that a cAMP-responsive kinase is activated when merozoites are transferred to EC buffer (Fig. 3A).

We used a molecular genetic approach to explore the regulatory role of PfPKA in microneme secretion. The *P. falciparum* transgenic line PHL dhfr-PfPKAr over-expresses the regulatory subunit (PfPKAr) (11, S4 Figure). Overexpression of PfPKAr results in reduced PKA activity and a parasite growth defect, which is restored by addition of the non-hydrolyzable cAMP analog dibutryl-cAMP (DiB) [11]. In contrast to wild type *P. falciparum* merozoites (Fig. 3A), lysates of *P. falciparum* PHL dhfr-PfPKAr merozoites in EC buffer do not phosphorylate kemptide at higher levels compared to lysates made in IC buffer (Fig. 3B). Treatment of *P. falciparum* PHL dhfr-PfPKAr merozoite lysates with DiB increases kemptide phosphorylation (Fig. 3B). These observations indicate that kemptide phosphorylation is primarily due to PfPKA and PfPKA activity increases when merozoites are transferred from IC to EC buffer.

Next, we followed secretion of PfAMA1 upon transfer of *P. falciparum* PHL dhfr-PfPKAr merozoites from IC to EC buffer with and without DiB treatment (Fig. 3C). PfAMA1 is not secreted when *P. falciparum* PHL dhfr-PfPKAr merozoites are transferred from IC to EC buffer (Fig. 3C). However, PfAMA-1 secretion is restored when *P. falciparum* PHL dhfr-PfPKAr merozoites are treated with DiB in EC buffer presumably due to the activation of PfPKA (Fig. 3C). These observations suggest that PfPKA plays a role in regulation of microneme secretion. Impairment of microneme secretion due to PfPKAr overexpression may be one of the reasons for poor growth of *P. falciparum* PHL dhfr-PfPKAr parasites.

### Crosstalk between cAMP and Ca^2+^ signaling in *P. falciparum* merozoites and regulation of microneme secretion

**Figure 4:**
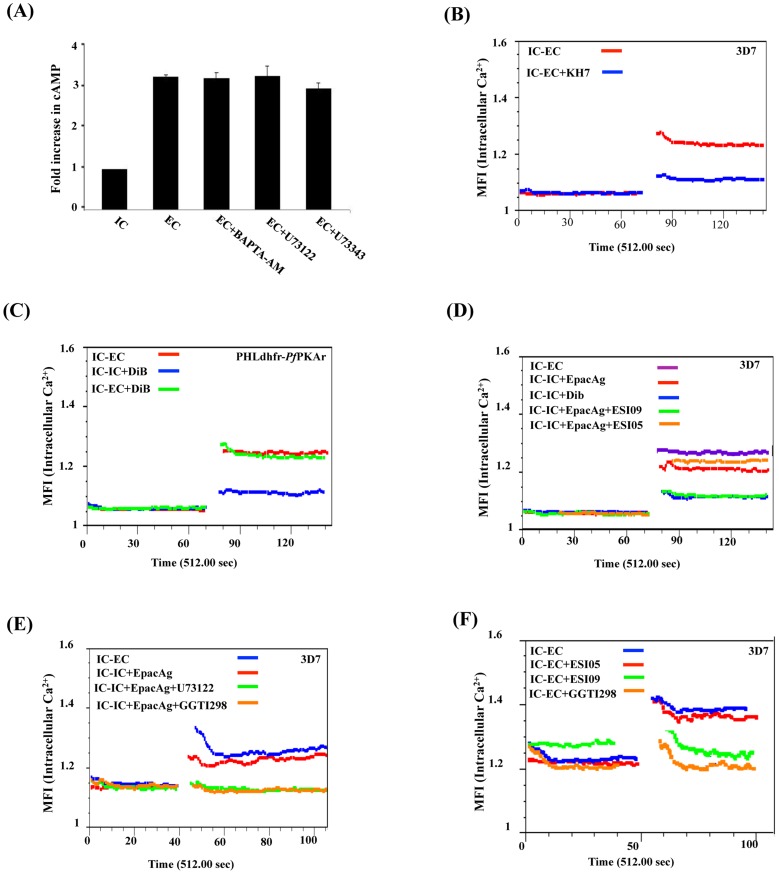
Crosstalk between cAMP and Ca^2+^ in *P. falciparum* merozoites. A) Ca^2+^ does not regulate cytosolic cAMP levels. *P. falciparum* merozoites were transferred from IC to EC buffer with or without treatment with BAPTA-AM or U73122. Levels of cytosolic cAMP were measured in merozoite lysates before and after transfer to EC buffer. Fold changes in cAMP levels per µg of merozoite protein (mean ± SD from 3 independent experiments) in different conditions relative to cAMP levels in IC buffer (mean ± SD) from 3 independent experiments are reported. Treatment of merozoites with BAPTA-AM or U73122 does not have any effect on rise in intracellular cAMP levels following transfer from IC to EC buffer. B) Rise in cytosolic Ca^2+^ is inhibited by ACβ inhibitor KH7. *P. falciparum* merozoites were loaded with Fluo-4AM and transferred from IC to EC buffer with or without treatment with KH7. Cytosolic Ca^2+^ levels in *P. falciparum* merozoites were measured before and after transfer by flow cytometry. Treatment with KH7 inhibits the rise in cytosolic Ca^2+^ following transfer to EC buffer. C) PKA does not regulate cytosolic Ca^2+^. *P. falciparum* PHL dhfr-PfPKAr merozoites were loaded with Fluo-4AM and transferred from IC to EC buffer or from IC to IC+DiB or from IC to EC+DiB. Cytosolic Ca^2+^ levels rise normally following transfer of *P. falciparum* merozoites from IC to EC buffer. Cytosolic Ca^2+^ levels do not rise when *P. falciparum* PHL dhfr-PfPKAr merozoites in IC buffer are treated with DiB indicating that PKA does not play a role in regulating Ca^2+^ levels in merozoites. D) Regulation of cytosolic Ca^2+^ levels in *P. falciparum* 3D7 merozoites by Epac. *P falciparum* merozoites loaded with Fluo-4AM were transferred from IC to EC buffer or from IC to IC buffer containing Epac agonist 8-Pcpt-2’-O-Me-cAMP (IC+Epac agonist), DiB (IC+DiB), or Epac agonist and Epac inhibitors (IC+Epac agonist+ESI-09 or IC+Epac agonist+ESI-05). Cytosolic Ca^2+^ levels rise when merozoites are transferred from IC to EC buffer, or IC buffer to IC+Epac agonist, but not when they are transferred from IC buffer to IC+DiB. EPAC1 antagonist ESI-09 inhibits rise in Ca^2+^ stimulated by Epac agonist. E) Regulation of cytosolic Ca^2+^ levels in *P. falciparum* 3D7 merozoites by Epac agonist, PLC inhibitor and Rap1 inhibitor. *P falciparum* merozoites loaded with Fluo-4AM were transferred from IC to EC buffer, or from IC to IC buffer containing Epac agonist (IC+Epac agonist), or IC buffer containing Epac agonist and PLC inhibitor (IC+Epac agonist+U73122), or IC buffer containing Epac agonist and Rap1 inhibitor GGTI298 (IC+Epac agonist+GGTI298). Cytosolic Ca^2+^ levels rise when merozoites are transferred from IC to EC buffer, or from IC to IC+Epac agonist. PLC inhibitor U73122 and Rap1 inhibitor GGTI298 inhibit rise in cytosolic Ca^2+^ stimulated by Epac agonist. F) Cytosolic Ca^2+^ levels in *P. falciparum* 3D7 merozoites following transfer to EC buffer in presence of Epac and Rap1 inhibitors. *P falciparum* merozoites loaded with Fluo-4AM were transferred from IC to EC buffer containing Epac inhibitors (EC+ESI-09 or EC+ESI-05), or EC buffer containing Rap1 inhibitor (EC+GGTI298). Cytosolic Ca^2+^ rises when merozoites are transferred from IC to EC buffer. Presence of Epac inhibitor ESI-09 and Rap1 inhibitor GGTI298 inhibits rise in cytosolic Ca^2+^.

Changes in the levels of cytosolic Ca^2+^ and cAMP control a variety of functions in diverse cell types [31]. We examined the crosstalk between these two ubiquitous second messengers in extracellular *P. falciparum* merozoites. We found that treatment of merozoites with the Ca^2+^ chelator, BAPTA-AM, or PLC inhibitor, U73122, does not affect the increase in cAMP levels when merozoites are transferred from IC to EC buffer (Fig. 4A). In contrast, treatment of merozoites with the ACβ inhibitor KH7 inhibits the increase in cytosolic Ca^2+^ levels when merozoites are transferred from IC to EC buffer (Fig. 4B) indicating that cAMP plays a key role in regulating cytosolic Ca^2+^ levels in *P. falciparum* merozoites.

Next, we found that Ca^2+^ levels increase in transgenic *P. falciparum* PHL dhfr-PKAr merozoites following their transfer from IC to EC buffer (Fig. 4B). Moreover, treatment of transgenic merozoites with DiB in IC buffer, which activates PfPKA, does not lead to further increase in cytosolic Ca^2+^ levels (Fig. 4C). These observations indicate that PfPKA does not play a role in generation of free cytosolic Ca^2+^ in *P. falciparum* merozoites following transfer from IC to EC buffer.

**Figure 5:**
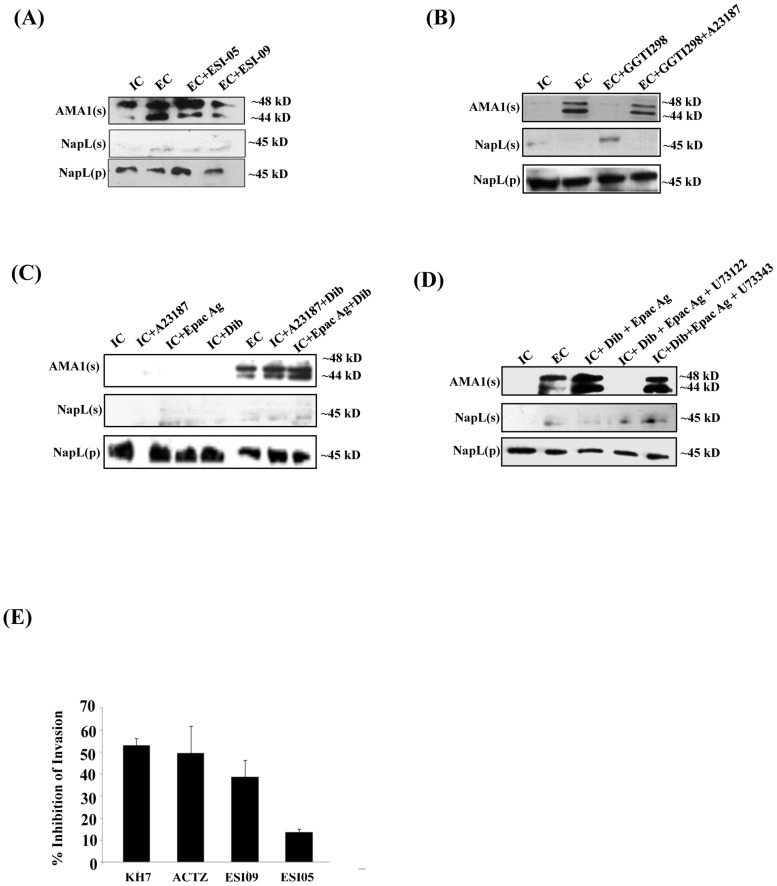
Role of Epac and PKA in regulation of microneme secretion and erythrocyte invasion by *P. falciparum* merozoites. *P. falciparum* merozoites were transferred from IC to EC buffer with or without treatment with various inhibitors. Secretion of PfAMA1 into merozoite supernatants (AMA1(s)) was detected by Western blotting. Cytoplasmic protein PfNapL was detected in *P. falciparum* merozoite supernatants (NapL(s)) and pellets (NapL(p)) by Western blotting under different conditions to control for merozoite lysis and number of merozoites used, respectively. A) Epac inhibitor ESI-09 blocks microneme secretion. Treatment of merozoites with 25 µM ESI-09 inhibits secretion of microneme protein PfAMA1 following transfer from IC to EC buffer. B) Rap1 inhibitor GGTI298 blocks microneme secretion. Treatment of merozoites with GGTI298 inhibits secretion of PfAMA1 following transfer from IC to EC buffer. Treatment of merozoites with GGTI298 in presence of A23187 does not block PfAMA1 secretion. C) Inhibition of microneme secretion by Epac and PKA pathway blockade. *P. falciparum* merozoites in IC buffer were treated with Ca^2+^ ionophore A23187 (IC+A23187), Epac agonist 8-Pcpt-2’-O-Me-cAMP (IC+Epac Ag) or dibutryl-cAMP (IC+DiB), or with various combinations (IC+A23187+DiB or IC+Epac Ag+DiB). Treatment of merozoites in IC buffer with Epac Ag and DiB (IC+Epac Ag+DiB) or A23187 and DiB (IC+A23187+DiB) triggers microneme secretion. D) *P. falciparum* merozoites in IC buffer were treated with dibutryl-cAMP and Epac agonist (IC+Dib+Epac agonist) or DiB+Epac Ag+PLC inhibitor U73122 or its inactive analog U73343. Treatment with U73122 inhibits microneme secretion induced by treatment with Dib and Epac Ag. E) Effect of various inhibitors of cAMP and Ca^2+^ signaling pathways on erythrocyte invasion by *P. falciparum* merozoites. *P. falciparum* 3D7 merozoites were isolated and allowed to invade erythrocytes in the presence of KH7 (50 µM), ACTZ (100 µM), ESI-09 (25 µM) and ESI-05 (25 µM). Newly invaded trophozoites were stained with ethidium bromide and scored by flow cytometry. Merozoites were allowed to invade erythrocytes in the absence of inhibitors as control. Percent invasion inhibition rates in presence of inhibitors are reported. Data represent mean (± SD) from three independent experiments with SD.

Alternative cAMP-responsive effectors, Epac 1 and Epac 2, have been shown to activate phospholipase C (PLC) in presence of cAMP leading to rise in cytosolic Ca^2+^ in mammalian cells [17], [32], [33]. *P. falciparum* codes for a single putative parasite PfEpac (PF3D7_1417400). Treatment of merozoites in IC buffer with the Epac agonist, 8-pCPT-2’-O-Me-cAMP [34], leads to a rise in cytosolic Ca^2+^ levels (Fig. 4D). This increase in Ca^2+^ is blocked if merozoites are treated with ESI-09, which inhibits both mammalian Epac 1 and Epac 2 [35] (Fig. 4D). ESI-05, which only inhibits mammalian Epac 2 [36], does not block increase in cytosolic Ca^2+^ following treatment with the Epac agonist (Fig. 4D). In mammalian cells, Epac activated by cAMP catalyzes the transfer of GTP to Rap1 [17]. Rap1-GTP activates PLC and triggers a rise in cytosolic Ca^2+^ through the PLC pathway [17]. Stimulation of merozoites with the Epac agonist in presence of GGTI298 [37], [38], which disrupts Rap1 activity, or the PLC inhibitor, U73122 [5], [39], inhibits rise in cytosolic Ca^2+^ (Fig. 4E). Moreover, treatment of merozoites with either ESI-09 or GGTI298 prior to transfer from IC to EC buffer also blocks rise in cytosolic Ca^2+^ (Fig. 4F) and inhibits PfAMA1 secretion (Fig. 5A, 5B). In contrast, ESI-05, which does not inhibit increase in cytosolic Ca^2+^ levels (Fig. 4F), does not block secretion of PfAMA1 (Fig. 5A). Treatment of merozoites with GGT1298 together with the Ca^2+^ ionophore A23187 restores microneme secretion (Fig. 5B). Thus, a cAMP-responsive Epac-Rap1 pathway is likely to be involved in regulating Ca^2+^ levels in *P. falciparum* merozoites.

To confirm the role of cAMP-responsive effectors PfPKA and PfEpac in microneme secretion, we treated merozoites in IC buffer with the Epac agonist, the Ca^2+^ ionophore A23187 and DiB, either individually or in combination. Treatment of merozoites in IC buffer with Epac agonist, A23187 or DiB individually does not trigger PfAMA1 secretion (Fig. 5C). However, treatment of merozoites in IC buffer with A23187 and DiB, or Epac-agonist and DiB triggers PfAMA1 secretion (Fig. 5C). The PLC inhibitor U73122 blocks PfAMA1 secretion induced by treatment of merozoites in IC buffer with Epac-agonist and DiB (Fig. 5D). However, the inactive analog, U73343 has no inhibitory effect. These observations suggest that Epac-agonist triggers rise in Ca^2+^ through the PLC pathway. Moreover, both Ca^2+^ and cAMP surges are required to trigger the secretion of microneme proteins in free merozoites.

### Blockade of cAMP- & Ca^2+^-signaling and inhibition of erythrocyte invasion by *P. falciparum* merozoites

Erythrocyte invasion assays were performed with purified extracellular merozoites in the presence of inhibitors of cAMP- and Ca^2+^-mediated signaling pathways. Merozoites were treated with ACβ inhibitor KH7, the CA inhibitor ACTZ, or Epac inhibitors ESI-09 and ESI-05, and allowed to invade human erythrocytes. Successful invasion events were scored by flow cytometry using DNA intercalating dye ethidium bromide to identify infected erythrocytes. KH7, ACTZ and ESI-09 inhibit invasion (Fig. 5E) adding to the evidence that both cAMP and Ca^2+^-dependent signaling pathways play critical roles in the process of red cell invasion by *P. falciparum* merozoites.

## Discussion

We have previously demonstrated that exposure of merozoites to a low K^+^ environment, as found in blood plasma, triggers a rise in cytosolic Ca^2+^ through a PLC mediated pathway leading to microneme secretion [5]. Although it is clear that a rise in cytosolic Ca^2+^ plays a key role in regulating microneme release, the signal transduction mechanisms by which exposure of extra-erythrocytic merozoites to a low K^+^ environment leads to a rise in cytosolic Ca^2+^ were not known.

Here, we demonstrate the role of cAMP in regulating levels of cytosolic Ca^2+^ and microneme secretion in response to exposure of merozoites to a low K^+^ environment. The transmembrane channel linked expressed in Paramecium, which contains a putative K^+^ channel-like transmembrane domain fused to the adenylyl cyclase catalytic domain, produces cAMP when the organism is exposed to a low K^+^ environment [18]. A homolog of tmAC, referred to as ACα is expressed in blood stage Plasmodium parasites. However, deletion of the gene encoding *P. berghei* ACα does not have any deleterious effect on growth of blood stage parasites [20]. In contrast, deletion of the gene encoding *P. berghei* ACα impairs hepatocyte invasion by *P. berghei* sporozoites [20]. These observations suggest that the soluble adenylyl cyclase, ACβ may play a more significant role in regulation of cAMP levels in Plasmodium merozoites.

**Figure 6:**
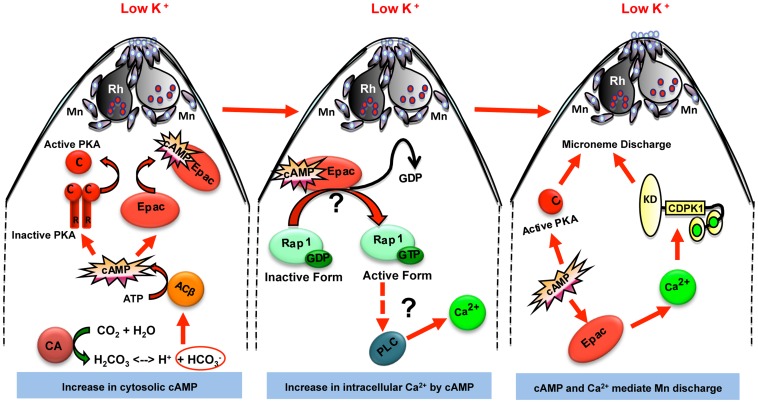
Model for cAMP and Ca^2+^ mediated signaling pathways that regulate microneme secretion in *P falciparum* merozoites. Exposure of *P. falciparum* merozoites to low K^+^ environment as present in blood plasma leads to production of H^+^ and HCO_3_^−^ ions by carbonic anhydrase (CA) to maintain pH. HCO_3_^−^ ions activate cytoplasmic adenylyl cyclase (ACβ) leading to rise in cytosolic levels of cAMP. Elevation of cAMP activates its downstream effectors PKA and Epac. Epac activates Rap1 by transferring GTP. Rap1-GTP activates PLC leading to rise in cytosolic Ca^2+^ levels, which leads to activation of calcium dependent protein kinase 1 (CDPK1) and calcium dependent phosphatase, calcineurin (CN), both of which directly play roles in microneme secretion. Mn, micronemes; Rh, rhoptries.

Here, we have used a series of pharmacological inhibitors to explore the role of PfACβ as well as other players involved in signaling pathways that control microneme secretion. Based on the observations made here, we propose the following model for the regulation of microneme secretion in response to environmental signals (Fig. 6). We propose that when merozoites are exposed to a low K^+^ environment, PfCA produces HCO_3_^−^ and H^+^ ions to maintain pH_i_ homeostasis (Fig. 6). Given the lack of information about the nature of ion channels present on the surface of *P. falciparum* merozoites, it is difficult to develop a molecular model to explain the activation of PfCA for maintenance of pH_i_ leading to production of HCO_3_^−^ when merozoites are exposed to a low K^+^ environment. Production of HCO_3_^−^ by PfCA activates PfACβ leading to a surge in cAMP levels in free merozoites (Fig. 6). Treatment of merozoites with CA inhibitor, ACTZ, or PfACβ inhibitor, KH7, blocks production of cAMP and inhibits microneme secretion. cAMP thus serves as a key second messenger that regulates microneme secretion in free merozoites during red cell invasion (Fig. 6).

Next, we demonstrated that production of cAMP in merozoites leads to activation of the cAMP-responsive kinase, PfPKA. We confirmed using a genetic approach that PfPKA plays a role in microneme secretion. Over-expression of the regulatory subunit, PfPKAr, inhibits PfPKA activity and blocks microneme secretion implicating PfPKA in regulation of microneme secretion. PfPKA has been shown to phosphorylate the cytoplasmic domain of PfAMA1 during invasion [40], although the precise functional role in the invasion process is not yet known. A global study of changes in protein phosphorylation in merozoites following their transfer to a low K^+^ environment is needed to identify the signaling pathways involved in microneme secretion. Determination of changes in protein phosphorylation in the presence and absence of PfPKA inhibition will identify substrates of PfPKA that may be involved in microneme secretion and invasion.

Given the essential roles that both Ca^2+^ and cAMP play in regulating microneme secretion in free merozoites, we examined the crosstalk between these key second messengers. Inhibiting rise in cytosolic Ca^2+^ by treatment of merozoites with BAPTA-AM or PLC inhibitor U73122 does not affect rise in cAMP when merozoites are exposed to a low K^+^ environment. In contrast, blocking a rise in cAMP with KH7 does inhibit increase in cytosolic Ca^2+^ levels. Thus, a rise in cAMP levels precedes and is necessary for an increase in cytosolic Ca^2+^ when merozoites are exposed to a low K^+^ environment.

We explored the mechanism by which cAMP regulates cytosolic Ca^2+^ levels in merozoites and found that PfEpac, rather than PfPKA, is responsible. We present pharmacological evidence to indicate that merozoites possess functional homologs of Epac and Rap1 that activate PLC to trigger a rise in cytosolic Ca^2+^ in response to increase in cAMP levels. A potential PfEpac is encoded by PF3D7_1417400 (www.plasmodb.org). A search for Rap1 homologs identifies a number of *P. falciparum* genes predicted to encode small GTPases. It remains to be seen which one these is the functional homolog of Rap1 that activates PLC in response to rise in cAMP in *P. falciparum* merozoites.

Previous studies with mature schizonts demonstrated that activation of a cGMP-dependent protein kinase, PfPKG, triggers release of proteins from micronemes and exonemes in merozoites leading to egress [41]. Secretion of exoneme proteins such as the protease PfSUB1 plays a critical role in merozoite egress from infected erythrocytes [42], [43]. The external, or internal signals that trigger a rise in cGMP and activate PfPKG leading to egress are not known. A sharp rise in cytosolic Ca^2+^ in intra-erythrocytic merozoites is also observed just prior to release of microneme and exoneme proteins and parasite egress [5], [43], [44]. The pathways for crosstalk between cGMP and Ca^2+^ in *P. falciparum* merozoites during egress have not been defined and whether cGMP-dependent PfPKG plays a role in microneme secretion in free merozoites after egress is not known. It will be important to understand the crosstalk between cGMP, cAMP and Ca^2+^ in merozoites leading to apical organelle exocytosis both before and after egress.

One possible mechanism for such crosstalk may be through phosphorylation of the PfPKAr subunit by PfPKG that would relieve inhibition of the catalytic PfPKAc subunit. PfPKAr displays characteristics of both mammalian RI and RII subunits [12]. Mammalian RI and RII subunits have distinct inhibitor sites. PfPKAr has a serine in the P-site typical of RII subunits and is phosphorylated by PfPKA *in vitro* [12]. However, PfPKAr also has a serine in the P+2 site typical of RI subunits. In mammalian cells this site is phosphorylated by PKG [45]. Like the P-site, the P+2 site in PfPKAr is also phosphorylated *in vivo* in *P. falciparum* schizonts [46]. Moreover, PfPKG can phosphorylate PfPKAr *in vitro* (Baker D. and Langsley G., unpublished data). Thus, PfPKG phosphorylation of PfPKAr could provide a mechanism for crosstalk between cGMP- and cAMP-mediated signaling.

A recent study describes a different mechanism for elevation of cytosolic Ca^2+^ in merozoites leading to microneme release [47]. This study suggests that interaction of the rhoptry neck protein PfRH1 with its erythrocyte receptor can trigger a surge in cytosolic Ca^2+^ levels in merozoites leading to release of microneme proteins such as EBA175 [47]. It remains to be seen if cAMP levels also rise and play a role in regulating Ca^2+^ levels following receptor-engagement by PfRH1. Moreover, the observation that PfRH1 knock-out parasites invade erythrocytes efficiently and display normal blood stage growth [48] suggests that *P. falciparum* merozoites must have alternative signaling mechanisms to trigger microneme release. Whether other members of the PfRH family such as PfRH2a/b can also mediate rise in cytosolic Ca^2+^ and microneme release remains to be determined. Alternatively, as described here, exposure to low K^+^ as found in blood plasma may provide such an alternative mechanism to trigger rise in cytosolic Ca^2+^ and microneme release.

Our study demonstrates that cAMP plays a central role in regulating microneme secretion during invasion (Fig. 6). Our data provides evidence to suggest that cAMP not only regulates microneme secretion by activating PfPKA, but it also regulates cytosolic Ca^2+^ levels by activation of PfEpac. Our definition of the signal transduction pathways leading to rise in cAMP and Ca^2+^ levels in response to external signals and the identification of cAMP and Ca^2+^ responsive effectors provides multiple points for therapeutic intervention. For example, inhibition of PfCA, PfACβ and PfEpac blocks microneme secretion and red blood cell invasion by *P. falciparum* merozoites. Small molecule inhibitors that specifically target these key-signaling molecules may provide valuable leads for the development of novel anti-malarial drugs that block blood stage growth of malaria parasites.

## Materials and Methods

### *In vitro* parasite culture

*P. falciparum* 3D7 was cultured in complete RPMI (RPMI 1640 (Invitrogen, USA), 27.2 mg/L hypoxanthine (Sigma, USA), 0.5 gms/L Albumax I (Gibco, USA) and 2 gm/L sodium bicarbonate (Sigma, USA) using O^+^ human erythrocytes under mixed gas (5% O_2_, 5% CO_2_ and 90% N_2_) as described previously [49]. *P. falciparum* PHL dhfr-*Pf*PKAr was cultured in complete RPMI according to the protocol described previously [11].

### Generation of PfACβ specific antisera

A peptide (1916–1930 aa) derived from PfACβ (PlasmoDB ID PF3D7_0802600) was synthesized, conjugated to keyhole limpet hemocyanin (KLH) and used to immunize mice to obtain anti-PfACβ specific antibodies.

### Generation of PfPKAr and PfPKAc specific antisera

The coding sequence of PfPKAr was amplified by PCR from *P. falciparum* cDNA with primers containing BamHI and XhoI restriction sites. The fragment was inserted into pCR2.1-TOPO and verified by sequencing. After digestion by XhoI and BamHI the insert was cloned into pGEX6P-1 (GE Healthcare) and transformed into *E. coli* BL21 that were grown in 50 mL of LB medium containing ampicillin (100 µg/mL, Euromedex). Recombinant protein expression was induced at 30°C for 4 h by addition of 0.2 mM IPTG (Euromedex). Cells were collected by centrifugation and lysed by sonication in lysis buffer (1× PBS-1 mM salt, 1% Triton, EDTA, protease inhibitors). The lysate was centrifuged (15000 rpm, 4°C, 60 min) and the soluble fraction incubated with glutathione sepharose beads (GE Healthcare) with stirring at 4°C for 90 min. Beads were washed with buffer (1× PBS, 1% Triton, 1 mM EDTA), the GST-tagged recombinant protein was eluted using increasing concentrations of glutathione. Purified recombinant antigen was used to immunize two rats to obtain anti-PfPKAr specific antibodies.

PfPKAc was cloned in the same way as PfPKAr. To increase stability of the recombinant catalytic subunit, we co-expressed PfPKAc-His with PfPKAr-GST in *E. coli* BL21. Transformed bacteria were grown in 50 mL of LB medium containing ampicillin (100 µg/mL, Euromedex). Recombinant protein expression was induced at 37°C for 4 h by addition of 0.5 mM IPTG (Euromedex). The bacterial pellet was washed once with 4 volumes of PBS and lysed using 4 volumes of Tris 20 mM at 4°C. After sonication, the lysate was centrifuged (6000 g, 4°C, 15 min) and the pellet resuspended in 3 volumes of inclusion body wash solution (2 M Urea, 500 mM NaCl, 2% NP40, 20 mM Tris-HCl, pH 8.0). The inclusion bodies were solubilised (5 mM Imidazole, 1 mM β-mercaptoethanol, 6 M Guanidine-HCl 6M, 20 mM Tris-HCl pH 8.0) and recombinant protein purified by metal affinity chromatography. His-tagged PfPKAc was eluted (8 M Urea to 0 M Urea, 20 mM imidazole to 500 mM imidazole, 500 mM NaCl, 1 mM β-mercaptoethanol, 20 mM Tris-Hcl pH 8.0) and used to immunize two rabbits to obtain anti-PfPKAc specific antibodies.

### Isolation of *P. falciparum* merozoites

*P. falciparum* blood stage cultures were synchronized with 5% sorbitol for at least two successive cycles. Synchronized *P. falciparum* cultures were used to isolate merozoites as described previously [5], [43]. Briefly, when majority of infected erythrocytes reached mature schizont stage with prominent segmentation of merozoites, the culture was resuspended in IC buffer (IC buffer – 140 mM KCl, 5 mM NaCl, 1 mM MgCl_2_, 5.6 mM glucose, 25 mM HEPES, pH 7.2) and schizonts were allowed to rupture to release merozoites over one hour at 37°C. Unruptured scizonts and uninfected erythrocytes were separated by centrifugation at 500 g for 5 min. The supernatant containing free merozoites was centrifuged at 3300 g for 5 min to collect merozoites. The merozoites were resuspended in IC buffer. The purity of merozoite preparations was confirmed by Giemsa staining (S5 Figure). Viability of merozoite preparations was estimated by labeling merozoites with dihydroethidine. Fluorescent merozoites were scored by flow cytometry and viability of merozoite preparations was estimated to be ∼65%.

### Drug treatments

*P. falciparum* merozoites were isolated in IC buffer and treated for 15 mins with the following at concentrations shown: 100 µM IBMX (3-isobutyl-1-methylxanthine, Calbiochem, USA), 50 µM KH7 (Calbiochem, USA), 100 µM ACTZ (acetazolamide, Sigma, USA), 20 µM DiB-cAMP (adenosine 3,5 cyclic monophosphate, N^6^, O^2^- dibutyryl sodium salt, Calbiochem, USA), 10 µM Ca^2+^ ionophore A23187 (Calbiochem, USA), 50 µM BAPTA-AM (Calbiochem, USA), 10 µM U73122 (Calbiochem, USA), 10 µM U73343 (Calbiochem, USA), 200 µM Epac agonist (8-pCPT-2’-O-Me-cAMP, Sigma, USA), 25 µM Epac antagonist ESI-05 (Biolog, Germany), 25 µM Epac antagonist ESI-09 (Biolog, Germany) and 50 µM geranyl geranyl transferase inhibitor, GGTI 298, which disrupts Rap1 function. Merozoites were then transferred to EC buffer (5 mM KCl, 140 mM NaCl, 1 mM MgCl_2_, 2 mM EGTA, 5.6 mM glucose, 25 mM HEPES, pH 7.2) followed by either measurement of cytosolic cAMP and Ca^2+^ levels in merozoites or microneme secretion.

### Microneme secretion assay

*P. falciparum* merozoites were isolated in IC buffer (140 mM KCl, 5 mM NaCl, 1 mM MgCl_2_, 5.6 mM glucose, 25 mM HEPES, pH 7.2), incubated for 15 min at 37°C with or without various pharmacological inhibitors or agonists as described above and transferred to EC buffer (5 mM KCl, 140 mM NaCl, 1 mM MgCl_2_, 2 mM EGTA, 5.6 mM glucose, 25 mM HEPES, pH 7.2) or IC-K_low_ buffer (5 mM NaCl, 5 mM KCl, 135 mM choline-Cl, 1 mM EGTA, 5.6 mM glucose, 25 mM HEPES, pH 7.2) with or without pharmacological inhibitors or agonists for 15 min at 37°C. Merozoites were separated by centrifugation as described above. Merozoite supernatants and merozoite pellets were separated by SDS-PAGE on a 12% gel under reducing conditions. Microneme proteins, PfAMA1 and EBA175, were detected in merozoite supernatants by Western blotting using anti-PfAMA1 and anti-EBA175 rabbit sera, respectively. Rabbit sera against cytoplasmic protein PfNapL were used to detect PfNapL in supernatants and merozoite pellets to estimate cell lysis and confirm that similar amounts of merozoites were used for each condition, respectively. Horseradish peroxidase (HRP)-conjugated anti-rabbit IgG goat antibodies (Sigma, USA) were used as secondary antibodies and detected by enhanced chemi-luminescence using ECL-Prime Western Blotting Detection reagent (GE Healthcare).

### Measurement of intracellular cAMP levels

*P. falciparum* merozoites were lysed with 0.1 N HCl and clarified by centrifugation at 6000 g. Supernatants were used for measuring the cytosolic levels of cAMP using cAMP direct immunoassay kit (Calbiochem, USA) as per manufacturer’s protocol. The kit uses a polyclonal antibody to bind cAMP present in the sample in a competitive manner. A standard curve was generated using known amounts of cAMP standards provided in the kit. Quantitative measurement of cAMP was obtained from the equation derived from the standard curve. The total protein content in each merozoite lysate sample was determined using Pierce BCA Protein Assay Kit (Pierce, USA) based on bicinchoninic acid (BCA). The amount of cAMP per µg of protein in each merozoite lysate sample was determined and used to calculate fold increase in cAMP per µg of merozoite protein material in each sample compared to control (merozoites in IC buffer).

### Adenylyl cyclase activity assay

*P falciparum* merozoites were isolated in RPMI 1640 as described, washed twice with phosphate buffered saline (PBS) and resuspended in 50 mM Tris pH 7.5, 150 mM NaCl. Merozoites were lysed by repeated freeze-thaw cycles and the lysate was clarified by centrifugation at 6000 g for 1 min at 4°C. The supernatant was incubated with different concentrations of NaHCO_3_ (0 mM, 10 mM, 20 mM and 40 mM) in the presence of 10 mM MgCl_2_, 10 mM CaCl_2_, 5 mM ATP and 500 µM IBMX for 30 min at 30°C. The soluble adenylyl cyclase inhibitor KH7 (50 µM) was used as negative control. The reactions were stopped by the addition of 0.1 N HCl and the level of cAMP produced was measured using the cAMP direct immunoassay kit as described above.

### Protein kinase A activity assay

*P. falciparum* merozoites were isolated as described above and incubated with or without KH7 (50 µM) in IC buffer for 15 min prior to transfer to EC buffer. Merozoite pellets were collected by centrifugation at 3000 g and used immediately to measure cAMP-dependent protein kinase A (PKA) activity. Merozoites were resuspended in ice-cold TNET buffer (50 mM Tris 7.4, 150 mM NaCl, 1 mM EDTA, and 1% Triton, protease inhibitor cocktail (Pierce, USA), 50 mM Na_3_VO_4_, 50 mM NaF) and lysed by repeated freeze-thaw cycles (4 cycles). The lysates were cleared by centrifugation at 6000 g for 15 min at 4°C. Total amount of protein in the supernatant was determined by (bicinchoninic acid assay (BCA) (Pierce, USA) using known amounts of bovine serum albumin (BSA) as standard. PKA activity was measured by quantitating incorporation of ^32^P in kemptide, a known peptide substrate of PKA (L-R-R-A-S-L-G, Kemptide PKA peptide substrate, Promega, USA) as described previously [9].

### Measurement of intracellular pH in *P. falciparum* merozoites

Intracellular pH (pH_i_) of *P. falciparum* merozoites was measured using the pH-sensitive fluorescent dye BCECF (2′,7′-bis-(2-carboxyethyl)-5-(and-6)-carboxyfluorescein) [30], [50]. *P. falciparum* merozoites were isolated in RPMI 1640 and loaded with BCECF-AM (acetoxymethylester of BCECF, Invitrogen, USA) for 20 min at 37°C in IC buffer. The merozoites were washed to remove excess dye and incubated with or without ACTZ in IC buffer at 37°C for 10 min. Merozoites were collected by centrifugation and resuspended in IC or EC buffer in the presence or absence of ACTZ and transferred to a 96 well microtiter plate (200 µL/well). Samples were excited monochromatically with λ_1_ = 440 nm and λ_2_ = 492 nm in succession for 0–10 minutes and emission was recorded at 535 nm on a fluorimeter (Perkin-Elmer Victor, USA). A quantitative measure of the pH_i_ is provided by the ratio of the fluorescence measured at 535 nm following excitation at 492 nm and 440 nm. For each experiment, a pH calibration curve was prepared by resuspending merozoites in buffer containing 130 mm KCl, 25 mM HEPES, 20 mM glucose and 1 mM MgCl_2_ with a pH range of 6.6 to 8.0 followed by the addition of 10 µm nigericin (Sigma). Nigericin equilibrates the intracellular and extracellular pH. The equation derived from the linear regression curve obtained by plotting the fluorescence ratio at different pH values (S6 Figure) was used to calculate pH_i_ of merozoites in different conditions.

### Measurement of cytosolic Ca^2+^ levels in *P. falciparum* merozoites

*P. falciparum* merozoites were isolated as described above, loaded with 10 µM Fluo-4-AM for 20 min at 37°C, washed, resuspended in IC buffer and used for experiments within 5 min, as described earlier [5], [51]. Fluo-4-AM loaded *P. falciparum* merozoites were treated with cAMP or Ca^2+^ modulating agents and transferred from IC to EC buffer. Fluorescence signal from Fluo-4 in merozoites under different conditions was measured by flow cytometry using FACSCalibur (Becton Dickinson, USA) and analyzed using CellQuest software. Briefly, Fluo-4-AM loaded merozoites were excited at 488 nm and fluorescence signal was detected with a 430 nm/30 nm band pass filter for periods of 2–3 mins. Merozoites were gated on the basis of their forward scatter and side scatter. The mean fluorescence intensity (MFI), which reflects cytosolic Ca^2+^ levels in merozoites, was plotted against time using FlowJo software.

### Erythrocyte invasion assay by *P. falciparum* merozoites

*P. falciparum* merozoites isolated as described above were either mock-treated or treated with 50 µm KH7 (Calbiochem, USA), 100 µm ACTZ (Sigma, USA), 25 µM ESI-05 (Biolog, Germany) or 25 µM ESI-09 (Biolog, Germany) for 15 min at 37°C, washed with IC buffer, resuspended in EC buffer and incubated with erythrocytes in EC buffer at 37°C under mixed gas environment for 2 h to allow invasion. EC buffer was then replaced with complete RPMI. After 18–20 h of incubation in complete RPMI under mixed gas environment to allow development of ring stages, the percentage of infected erythrocytes was scored by flow cytometry to determine invasion rates, as described earlier [52].

### Immunofluorescence microscopy

*P. falciparum* schizonts and merozoites were smeared on glass slides, dried and fixed with pre-chilled methanol. Smears were blocked with 3% BSA in 1× PBS for 2 h at room temperature (RT) and probed with anti-PfACβ mouse sera diluted 1∶100, followed by Alexa-Fluor 488 conjugated goat anti-mouse IgG antibody diluted 1∶200. For co-immuno-staining, smears were also probed with rabbit sera (1∶100 dilution) against the cytoplasmic protein PfNAPL [20]. After washing, smears were incubated with Alexa-Fluor 488-conjugated goat anti-mouse IgG (1∶200, Molecular Probes, USA) and Alexa-Fluor 594-conjugated goat anti-rabbit IgG (1∶200 dilution, Molecular Probes, USA), for 1 hr at RT. The slides were washed, mounted with 4′, 6-diamidino-2-phenylindole dihydrochloride (DAPI, Molecular Probes, USA) and antifade mounting media (Molecular Probes, USA) and analyzed using a Nikon A1-R confocal microscope.

## Supporting Information

S1 Figure **Detection of** ***P. falciparjum*** **adenylyl cyclase β (PfACβ) in** ***P. falciparum*** **merozoite and schizont lysates by western blotting.** *P falciparum* merozoite and schizont lysates were separated by SDS-PAGE and transferred to nitrocellulose. Presence of PfACβ was detected by Western blotting using mouse sera raised against a peptide (1916–1930 aa) derived from PfACβ (PlasmoDB ID PF3D7_0802600) conjugated to keyhole limpet hemocyanin (KLH). Native PfACβ was detected at ∼270 kDa in *P. falciparum* merozoite and schizont lysates. Pre-immune mouse serum was used as negative control. Merozoite lysates were also probed for the cytoplasmic protein, *P. falciparum* nucleosome assembly protein-L (PfNapL) and *P. falciparum* merozoite surface protein MSP-1 (PfMSP1) using anti-PfNapL rabbit sera and anti-PfMSP1_19_ rabbit sera respectively.
(TIFF)

S2 Figure **Inhibition of EBA175 secretion with KH7.** *P. falciparum* merozoites were transferred from IC to EC buffer with or without prior treatment with mammalian ACβ inhibitor KH7. Presence of EBA175 was detected in the merozoite supernatant by Western blotting. Mouse antisera were used to detect cytoplasmic protein NAPL in merozoite pellets as loading control and in supernatants to control for cell lysis. EBA175 is secreted when merozoites are transferred from IC to EC buffer. Secretion is blocked by prior treatment of merozoites with KH7.
(TIFF)

S3 Figure **Regulation of microneme secretion by treatment of** ***P. falciparum*** **merozoites with increasing concentrations of NaHCO_3_.** *P. falciparum* merozoites in IC buffer were transferred to IC buffer containing increasing concentrations of NaHCO_3_ (10 mM, 20 mM and 40 mM), EC buffer or EC buffer +40 mM NaHCO_3_ for 15 min at 37°C. Secretion of PfAMA1 into merozoite supernatants (AMA1(s)) was detected by Western blotting. Cytoplasmic protein PfNapL was detected in *P. falciparum* merozoite supernatants (NapL(s)) and pellets (NapL(p)) by Western blotting under different conditions to control for merozoite lysis and number of merozoites used, respectively. Treatment of merozoites with NaHCO_3_ triggers secretion of microneme protein PfAMA1 in IC buffer in a concentration dependent manner.
(TIFF)

S4 Figure **Expression of PKA regulatory subunit (PKAr) in** ***P. falciparum*** **schizonts.** A) Detection of PKAr in schizonts of *P. falciparum* lines PHL-dhfr-PKAr and *P. falciparum* PHL-dhfr-luciferase by Western blotting. Lysates of *P. falciparum* PHL-dhfr-luciferase and *P. falciparum* PHL-dhfr-PfPKAr schizonts were separated by SDS-PAGE, transferred to nitrocellulose and probed for presence of PfPKAr by Western blotting using rat anti-PfPKAr serum. Antisera raised against *P. falciparum* aldolase were used as loading control. The band intensity corresponding to PKAr was higher in PHL-PfPKAr schizont lysates in comparison to PHL-luciferase schizont lysates, whereas the reactivity of anti-Pfaldolase serum was similar confirming equal loading. The specificity of Western blot detection was confirmed by incubation of anti-PfPKAr serum with excess of recombinant PfPKAr prior to detection by Western blotting. B) Detection of PKAr in *P. falciparum* 3D7 merozoites by immunofluorescence assay (IFA). Mouse sera raised against PfPKAr and rabbit sera raised against PfMSP1_19_ were used in IFA to detect expression and localize PfPKAr and PfMSP1 respectively. There is significant overlap between PfPKAr and PfMSP indicating that a significant portion of PfPKAr is associated with the merozoite plasma membrane.
(TIFF)

S5 Figure **Viabilty and purity of** ***P. falciparum*** **merozoites.** Viabilty of *P. falciparum* merozoites was analyzed by staining with dihydroethidine (DHE). *P. falciparum* merozoites were incubated with DHE (10 µg/ml) for 20 min at 37°C after purification. A) DHE-stained merozoites were further stained with nuclear staining dye DAPI and visualized using a confocal Nikon A1R microscope. B) DHE-stained merozoites were also analyzed by flow cytometry using a FACS Calibur (Becton & Dickinson, USA). 100,000 RBCs were scored per sample for fluorescence staining with DHT to determine percentage of merozoites that are viable.
(TIFF)

S6 Figure **Standard curve for measurement of intracellular pH in** ***P falciparum*** **merozoites.** *P. falciparum* merozoites were loaded with pH sensitive fluorescent dye BCECF-AM, resuspended in buffer at 10 different pH conditions (6.4, 6.6, 6.8, 7.0, 7.2, 7.4, 7.6, 7.8, 8.0) and treated with nigericin for 10 min at 37°C to allow extracellular and intracellular pH to equilibrate. Samples were excited at 440 nm and 492 nm and mean fluorescent intensity (MFI) was measured at 535 nm. Ratio of MFI measured at 535 nm following excitation at 492 nm and 440 nm was plotted against pH to generate a standard curve.
(TIFF)

## References

[1] Cowman AF, Crabb BS (2006) Invasion of red blood cells by malaria parasites. Cell 124: 755–766. 16497586 10.1016/j.cell.2006.02.006

[2] Gaur D, Chitnis CE (2011) Molecular interactions and signaling mechanisms during erythrocyte invasion by malaria parasites. Curr Opin Microbiol 14: 422–428. 21803641 10.1016/j.mib.2011.07.018

[3] Sharma P, Chitnis CE (2013) Key molecular events during host cell invasion by Apicomplexan pathogens. Curr Opin Microbiol 16: 432–437. 23895827 10.1016/j.mib.2013.07.004

[4] Baum J (2013) A complete molecular understanding of malaria parasite invasion of the human erythrocyte: are we there yet? Pathog Glob Health 107: 107–110. 23683362 10.1179/2047772413Z.000000000121 PMC4003585

[5] Singh S, Alam MM, Pal-Bhowmik I, Brzostowski JA, Chitnis CE (2010) Distinct external signal trigger sequential release of apical organelles during erythrocyte invasion by malaria parasites. PLoS Pathog 6: e1000746. 20140184 10.1371/journal.ppat.1000746 PMC2816683

[6] Siddiqui FA, Dhawan S, Singh S, Singh B, Gupta P, et al (2013) A thrombospondin structural repeat containing rhoptry protein from *Plasmodium falciparum* mediates erythrocyte invasion. Cell Microbiol 15: 1341–56. 23387921 10.1111/cmi.12118

[7] Kim C, Xuong NH, Taylor SS (2005) Crystal structure of a complex between the catalytic and regulatory (RIalpha) subunits of PKA. Science 307: 690–696. 15692043 10.1126/science.1104607

[8] Read LK, Mikkelsen RB (1990) Cyclic AMP- and Ca^2+^ -dependent protein kinases in *Plasmodium falciparum* . Exp Parasitol 71: 39–48. 2191871 10.1016/0014-4894(90)90006-x

[9] Syin C, Parzy D, Traincard F, Boccaccio I, Joshi MB, et al (2001) The H89 cAMP-dependent protein kinase inhibitor blocks *Plasmodium falciparum* development in infected erythrocytes. Eur J Biochem 268: 4842–4849. 11559352 10.1046/j.1432-1327.2001.02403.x

[10] Li J, Cox LS (2000) Isolation and characterisation of a cAMP-dependent protein kinase catalytic subunit gene from *Plasmodium falciparum* . Mol Biochem Parasitol 109: 157–163. 10960174 10.1016/s0166-6851(00)00242-5

[11] Merckx A, Nivez MP, Bouyer G, Alano P, Langsley G, et al (2008) *Plasmodium falciparum* regulatory subunit of cAMP-dependent PKA and anion channel conductance. PLoS Pathogens 4: e19. 18248092 10.1371/journal.ppat.0040019 PMC2222956

[12] Haste NM, Talabani H, Doo A, Merckx A, Langsley G, et al (2012) Exploring the *Plasmodium falciparum* cyclic-adenosine monophosphate (cAMP)-dependent protein kinase (*Pf* PKA) as a therapeutic target. Microbes and Infection 14: 838–850. 22626931 10.1016/j.micinf.2012.05.004 PMC3967591

[13] Kurokawa H, Kato K, Iwanaga T, Sugi T, Sudo A, et al (2011) Identification of *Toxoplasma gondii* cAMP dependent protein kinase and its role in tachyzoite growth. PLoS One 2011 6(7): e22492 10.1371/journal.pone.0022492 PMC3140512 21799871

[14] Kirkman LA, Weiss LM, Kim K (2001) Cyclic nucleotide signaling in *Toxoplasma gondii* bradyzoite differentiation. Infect Immun 69(1): 148–53. 11119500 10.1128/IAI.69.1.148-153.2001 PMC97866

[15] Eaton MS, Weiss LM, Kim K (2006) Cyclic nucleotide kinases and tachyzoite-bradyzoite transition in *Toxoplasma gondii*. 2013. Int J Parasitol 36(1): 107–14. 16216248 10.1016/j.ijpara.2005.08.014 PMC3109623

[16] Hartmann A, Arroyo-Olarte RD, Imkeller K, Hegemann P, Lucius R, et al (2013) Optogenetic modulation of an adenylate cyclase in Toxoplasma gondii demonstrates a requirement of the parasite cAMP for host-cell invasion and stage differentiation. J Biol Chem 288 19: 13705–17 10.1074/jbc.M113.465583 23525100 PMC3650408

[17] Gloerich M, Bos JL (2010) Epac: defining a new mechanism for cAMP action. Annu Rev Pharmacol Toxicol 50: 355–375. 20055708 10.1146/annurev.pharmtox.010909.105714

[18] Weber JH, Vishnyakov A, Hambach K, Schultz A, Schiltz JE, et al (2004) Adenylyl cyclases from Plasmodium, Paramecium and Tetrahymena are novel ion channel/enzyme fusion proteins. Cell Signal 16: 115–125. 14607282 10.1016/s0898-6568(03)00129-3

[19] Salazar E, Bank EM, Ramsey N, Hess KC, Deitsch DW, et al (2012) Characterization of *Plasmodium falciparum* adenylyl cyclase β and its role in erythrocytic stage parasites. PLoS One 7: e39769. 22761895 10.1371/journal.pone.0039769 PMC3383692

[20] Ono T, Cabrita-Santos L, Leitao R, Bettiol E, Purcell LA, et al (2008) Adenylyl cyclase α and cAMP signaling mediate *Plasmodium* sporozoite apical regulated exocytosis and hepatocyte infection. PLoS Pathog 4 2: e1000008. 18389080 10.1371/journal.ppat.1000008 PMC2279260

[21] Chandra BR, Olivieri A, Silvestrini F, Alano P, Sharma A (2005) Biochemical characterization of the two nucleosome assembly proteins from *Plasmodium falciparum* . Mol Biochem Parasitol 142: 237–47. 15899528 10.1016/j.molbiopara.2005.04.006

[22] Howell SA, Withers-Martinez C, Kocken CH, Thomas AW, Blackman MJ (2001) Proteolytic processing and primary structure of *Plasmodium falciparum* apical membrane antigen-1. J Biol Chem 276 33: 31311–20. 11399764 10.1074/jbc.M103076200

[23] Beavo JA, Rogers NL, Crofford OB, Hardman JG, Sutherland EW, Newman EV (1970) Effects of xanthine derivatives on lipolysis and on adenosine 3’,5’-monophosphate phosphodiesterase activity. Mol Pharmacol 6: 597–603. 4322367

[24] Chen Y, Cann MJ, Litvin TN, Iourgenko V, Sinclair ML, et al (2000) Soluble adenylyl cyclase as an evolutionarily conserved bicarbonate sensor. Science 289: 625–628. 10915626 10.1126/science.289.5479.625

[25] Cann MJ, Hammer A, Zhou J, Kanacher T (2003) A defined subset of adenylyl cyclases is regulated by bicarbonate ion. J Biol Chem 278: 35033–35038. 12829712 10.1074/jbc.M303025200

[26] Kobayashi M, Buck J, Levin LR (2004) Conservation of functional domain structure in bicarbonate-regulated “soluble” adenylyl cyclases in bacteria and eukaryotes. Dev Genes Evol 214: 503–509. 15322879 10.1007/s00427-004-0432-2 PMC3644946

[27] Lindskog S (1997) Structure and mechanism of carbonic anhydrase. Pharmcol Ther 74: 1–20. 10.1016/s0163-7258(96)00198-2 9336012

[28] Krungkrai J, Krungkrai SR, Supuran CT (2008) Carbonic anhydrase inhibitor: Inhibition of *Plasmodium falciparun* carbonic anhydrase with aromatic/heterocyclic sulfonamides- in vivo and in vitro studies. Bioorg Med Chem Lett 18: 5466–5474. 18805693 10.1016/j.bmcl.2008.09.030

[29] Krungkrai J, Supuran CT (2008) The alpha carbonic anhydrase from malaria parasite and its inhibition. Curr Pharm Des 14: 631–640. 18336308 10.2174/138161208783877901

[30] Rink TJ, Tsien RY, Pozzan T (1982) Cytoplasmic pH and free Mg^2+^ in lymphocytes. J Cell Biol 95: 189–96. 6815204 10.1083/jcb.95.1.189 PMC2112339

[31] Borodinsky LN, Spitzer NC (2006) Second messenger pas de deux: the coordinated dance between calcium and cAMP. Sci STKE *pe22* 10.1126/stke.3362006pe22 16720840

[32] Hoque KM, Woodword OM, Rossum DB, Zachos NC, Chen L, et al (2009) Epac1 mediates protein kinase A-independent mechanism of forskolin activated intestinal chloride secretion. J Gen Physiol 135: 43–58. 10.1085/jgp.200910339 PMC2806414 20038525

[33] Purves GI, Kamishima T, Davies LM, Quayle JM, Dart C (2009) Exchange protein activated by AMP (Epac) mediates cAMP-dependent but protein kinase A-insensitive modulation of vascular ATP-sensitive potassium channels. J Physiol 587: 3639–3650. 19491242 10.1113/jphysiol.2009.173534 PMC2742287

[34] Enserink JM, Christensen AE, de Rooij J, van Triest M, Schwede F, et al (2002) A novel Epac-specific cAMP analogue demonstrates independent regulation of Rap1 and ERK. Nat Cell Biol 4 11: 901–6. 12402047 10.1038/ncb874

[35] Almahariq M, Tsalkova T, Mei FC, Chen H, Zhou J, et al (2013) A novel EPAC-specific inhibitor suppresses pancreatic cancer cell migration and invasion. Mol Pharmacol 83 1: 122–8. 23066090 10.1124/mol.112.080689 PMC3533471

[36] Tsalkova T, Mei FC, Li S, Chepurny OG, Leech CA, et al (2012) Isoform-specific antagonists of exchange proteins directly activated by cAMP. Proc Natl Acad Sci U S A 109: 18613–8. 23091014 10.1073/pnas.1210209109 PMC3494926

[37] Vogt A, Qian Y, McGuire TF, Hamilton AD, Sebti SM (1996) Protein geranylgeranylation, not farnesylation, is required for the G1 to S phase transition in mouse fibroblasts. 1996. Oncogene 13 9: 1991–9. 8934546

[38] Chakrabarti D, Da Silva T, Barger J, Paquette S, Patel H, Patterson S, Allen CM, et al Protein farnesyltransferase and protein prenylation in *Plasmodium falciparum* . J Biol Chem 277: 42066–73. 10.1074/jbc.M202860200 12194969

[39] Yule DI, Williams JA (1992) U73122 inhibits Ca2+ oscillations in response to cholecystokinin and carbochol but not to JMV-180 in rat pancreatic acinar cells. J Biol Chem 267: 13830–13835. 1629184

[40] Leykauf K, Treeck M, Gilson PR, Nebl T, Braulke T, et al (2010) Protein kinase A dependent phosphorylation of apical membrane antigen 1 plays an important role in erythrocyte invasion by the malaria parasite. PLoS Pathog 6: e1000941. 20532217 10.1371/journal.ppat.1000941 PMC2880582

[41] Collins CR, Hackett F, Strath M, Penzo M, Withers-Martinez C, et al (2013) Malaria parasite cGMP-dependent protein kinase regulates blood stage merozoite secretory organelle discharge and egress. PLoS Pathog 9: e1003344. 23675297 10.1371/journal.ppat.1003344 PMC3649973

[42] Yeoh S, O'Donnell RA, Koussis K, Dluzewski AR, Ansell KH, et al (2007) Subcellular discharge of a serine protease mediates release of invasive malaria parasites from host erythrocytes. Cell 131: 1072–1083. 18083098 10.1016/j.cell.2007.10.049

[43] Agarwal S, Singh MK, Garg S, Chitnis CE, Singh S (2012) Ca(2+) -mediated exocytosis of subtilisin-like protease 1: a key step in egress of *Plasmodium falciparum* merozoites. Cell Microbiol 15: 910–921. 23217145 10.1111/cmi.12086

[44] Glushakova S, Lizunov V, Blank PS, Melikov K, Humphrey G, et al (2013) Cytoplasmic free Ca2+ is essential for multiple steps in malaria parasite egress from infected erythrocytes. Malar J 30: 12–41. 10.1186/1475-2875-12-41 PMC3564835 23363708

[45] Geahlen RL, Krebs EG (1980) Regulatory subunit of the type I cAMP-dependent protein kinase as an inhibitor and substrate of the cGMP-dependent protein kinase. J Biol Chem 255: 1164–1169. 6243294

[46] Lasonder E, Treeck M, Alam M, Tobin AB (2012) Insights into the Plasmodium falciparum schizont phospho-proteome. Microbes Infect 14: 811–819. 22569589 10.1016/j.micinf.2012.04.008

[47] Gao X, Gunalan K, Yap SSL, Preiser P (2013) Triggers of key calcium signals during erythrocyte invasion by *Plasmodium falciparum* . Nature Commun 4: 2862 doi: 10.1038 24280897 10.1038/ncomms3862 PMC3868333

[48] Triglia T, Duraisingh MT, Good RT, Cowman AF (2005) Reticulocyte-binding protein homologue 1 is required for sialic acid dependent invasion into human erythrocytes by *Plasmodium falciparum* . Mol Microbiol 55: 162–174. 15612925 10.1111/j.1365-2958.2004.04388.x

[49] Trager W, Jensen JB (1976) Human malaria parasites in continuous culture. Science 193: 673–675. 781840 10.1126/science.781840

[50] Van der Heyden N, Benaim G, Docampo R (1996) The role of a H+-ATPase in the regulation of cytoplasmic pH in Trypanosoma cruzi epimastigotes. Biochem J 318: 103–109. 8761458 10.1042/bj3180103 PMC1217594

[51] Singh S, Chitnis CE (2013) Flow cytometry-based methods for measurement of cytosolic calcium and surface proteins expression in *Plasmodium falciparum* merozoites. Methods Mol Biol 923: 281–290. 22990785 10.1007/978-1-62703-026-7_19

[52] Sahar T, Reddy KS, Bharadwaj M, Pandey AK, Singh S, et al (2011) Plasmodium falciparum reticulocyte binding-like homologue protein 2 (PfRH2) is a key adhesive molecule involved in erythrocyte invasion. PLoS One 6: e17102. 21386888 10.1371/journal.pone.0017102 PMC3046117

