# Supplementary figures and images for: The Central Role of cAMP in Regulating Plasmodium falciparum Merozoite Invasion of Human Erythrocytes
Source: PLoS Pathog. 2014 Dec 18;10(12):e1004520. doi: 10.1371/journal.ppat.1004520 (PMC4270784; doi:10.1371/journal.ppat.1004520)

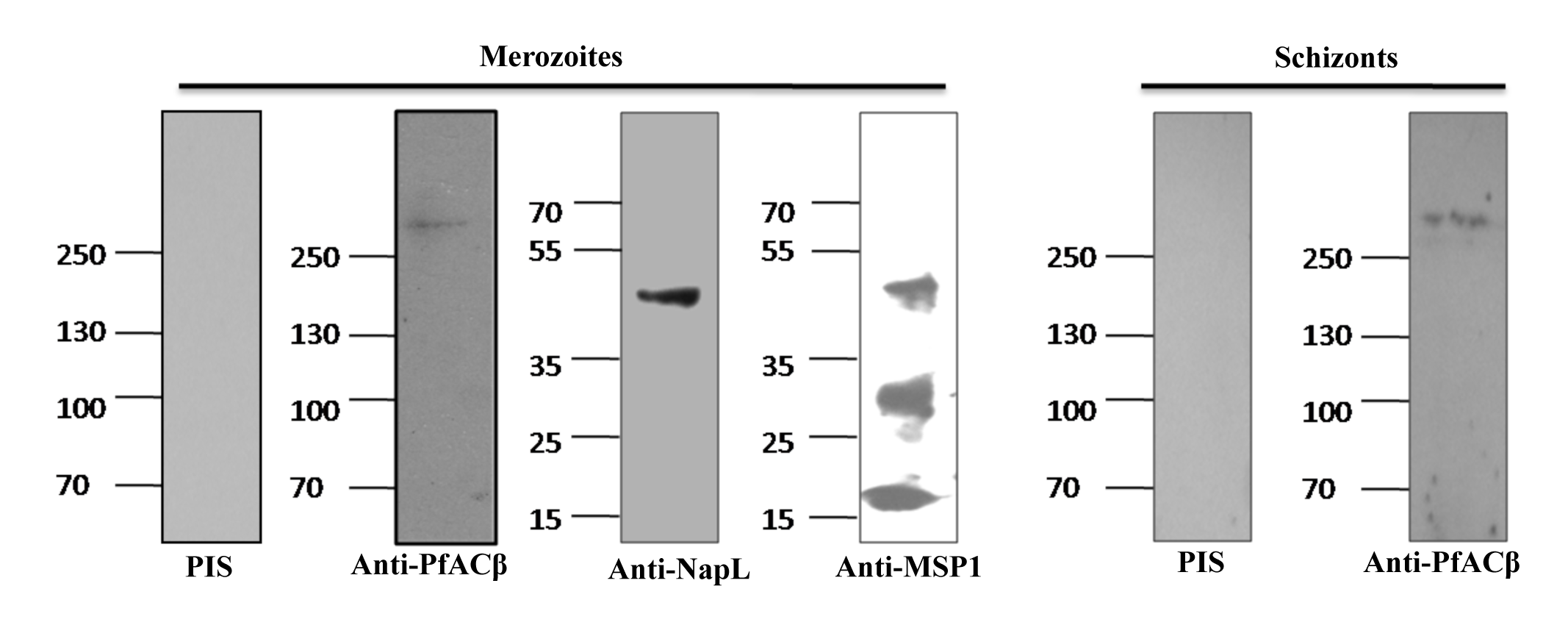

Supplement: S1 Figure — Detection of P. falciparjum adenylyl cyclase β (PfACβ) in P. falciparum merozoite and schizont lysates by western blotting. P falciparum merozoite and schizont lysates were separated by SDS-PAGE and transferred to nitrocellulose. Presence of PfACβ was detected by Western blotting using mouse sera raised against a peptide (1916–1930 aa) derived from PfACβ (PlasmoDB ID PF3D7_0802600) conjugated to keyhole limpet hemocyanin (KLH). Native PfACβ was detected at ∼270 kDa in P. falciparum merozoite and schizont lysates. Pre-immune mouse serum was used as negative control. Merozoite lysates were also probed for the cytoplasmic protein, P. falciparum nucleosome assembly protein-L (PfNapL) and P. falciparum merozoite surface protein MSP-1 (PfMSP1) using anti-PfNapL rabbit sera and anti-PfMSP119 rabbit sera respectively. (TIFF) [file ppat.1004520.s001.tiff]

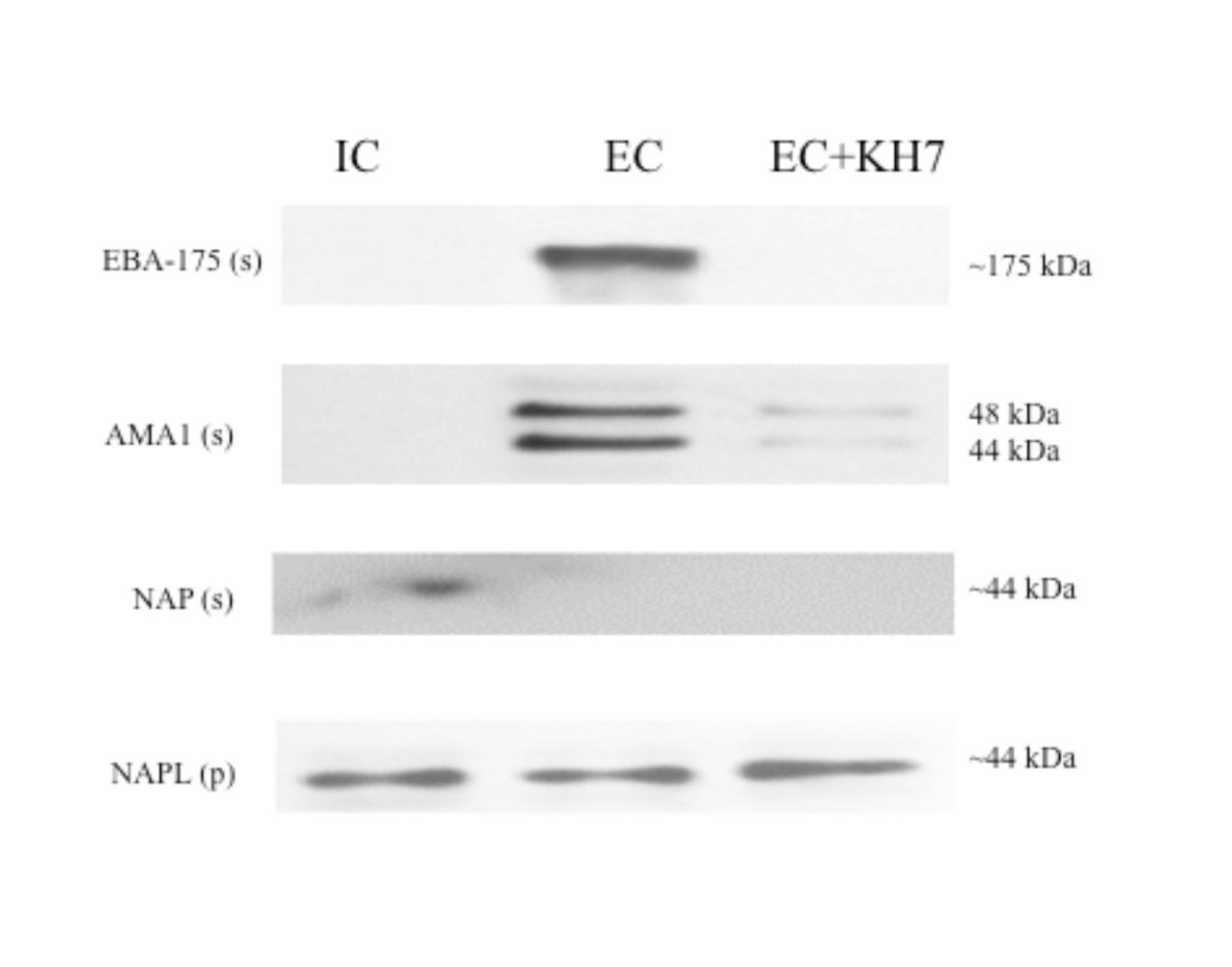

Supplement: S2 Figure — Inhibition of EBA175 secretion with KH7. P. falciparum merozoites were transferred from IC to EC buffer with or without prior treatment with mammalian ACβ inhibitor KH7. Presence of EBA175 was detected in the merozoite supernatant by Western blotting. Mouse antisera were used to detect cytoplasmic protein NAPL in merozoite pellets as loading control and in supernatants to control for cell lysis. EBA175 is secreted when merozoites are transferred from IC to EC buffer. Secretion is blocked by prior treatment of merozoites with KH7. (TIFF) [file ppat.1004520.s002.tiff]

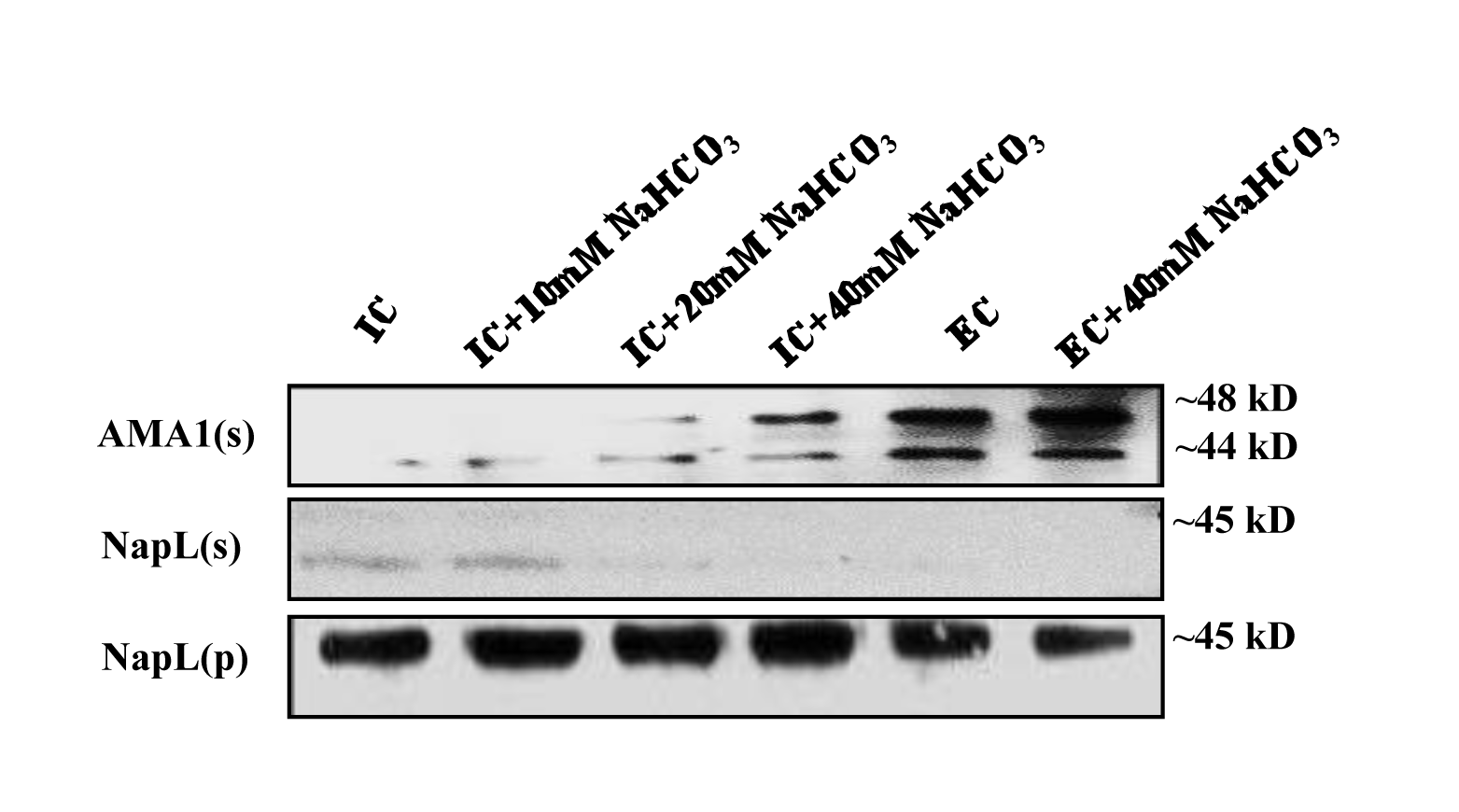

Supplement: S3 Figure — Regulation of microneme secretion by treatment of P. falciparum merozoites with increasing concentrations of NaHCO3. P. falciparum merozoites in IC buffer were transferred to IC buffer containing increasing concentrations of NaHCO3 (10 mM, 20 mM and 40 mM), EC buffer or EC buffer +40 mM NaHCO3 for 15 min at 37°C. Secretion of PfAMA1 into merozoite supernatants (AMA1(s)) was detected by Western blotting. Cytoplasmic protein PfNapL was detected in P. falciparum merozoite supernatants (NapL(s)) and pellets (NapL(p)) by Western blotting under different conditions to control for merozoite lysis and number of merozoites used, respectively. Treatment of merozoites with NaHCO3 triggers secretion of microneme protein PfAMA1 in IC buffer in a concentration dependent manner. (TIFF) [file ppat.1004520.s003.tiff]

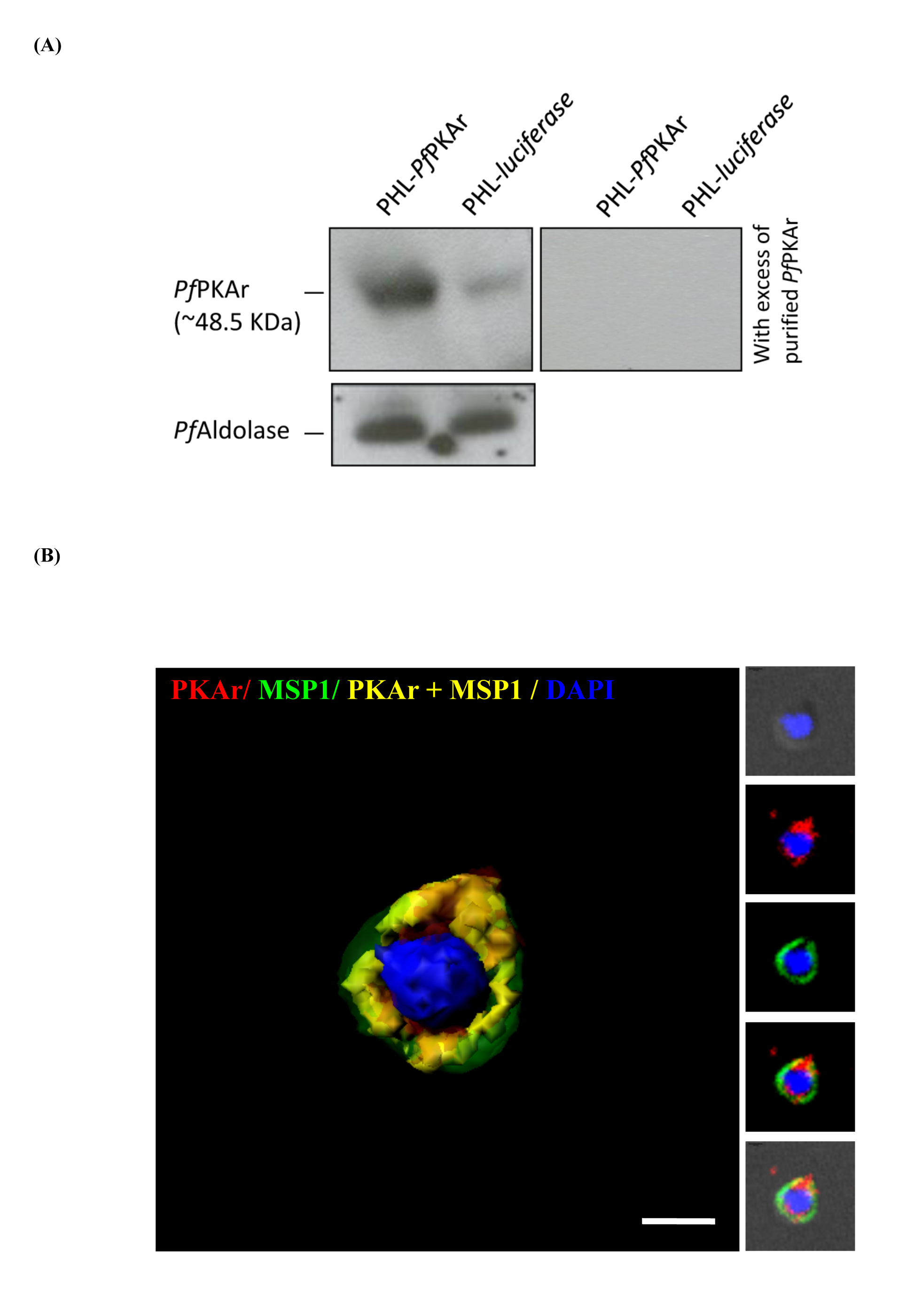

Supplement: S4 Figure — Expression of PKA regulatory subunit (PKAr) in P. falciparum schizonts. A) Detection of PKAr in schizonts of P. falciparum lines PHL-dhfr-PKAr and P. falciparum PHL-dhfr-luciferase by Western blotting. Lysates of P. falciparum PHL-dhfr-luciferase and P. falciparum PHL-dhfr-PfPKAr schizonts were separated by SDS-PAGE, transferred to nitrocellulose and probed for presence of PfPKAr by Western blotting using rat anti-PfPKAr serum. Antisera raised against P. falciparum aldolase were used as loading control. The band intensity corresponding to PKAr was higher in PHL-PfPKAr schizont lysates in comparison to PHL-luciferase schizont lysates, whereas the reactivity of anti-Pfaldolase serum was similar confirming equal loading. The specificity of Western blot detection was confirmed by incubation of anti-PfPKAr serum with excess of recombinant PfPKAr prior to detection by Western blotting. B) Detection of PKAr in P. falciparum 3D7 merozoites by immunofluorescence assay (IFA). Mouse sera raised against PfPKAr and rabbit sera raised against PfMSP119 were used in IFA to detect expression and localize PfPKAr and PfMSP1 respectively. There is significant overlap between PfPKAr and PfMSP indicating that a significant portion of PfPKAr is associated with the merozoite plasma membrane. (TIFF) [file ppat.1004520.s004.tiff]

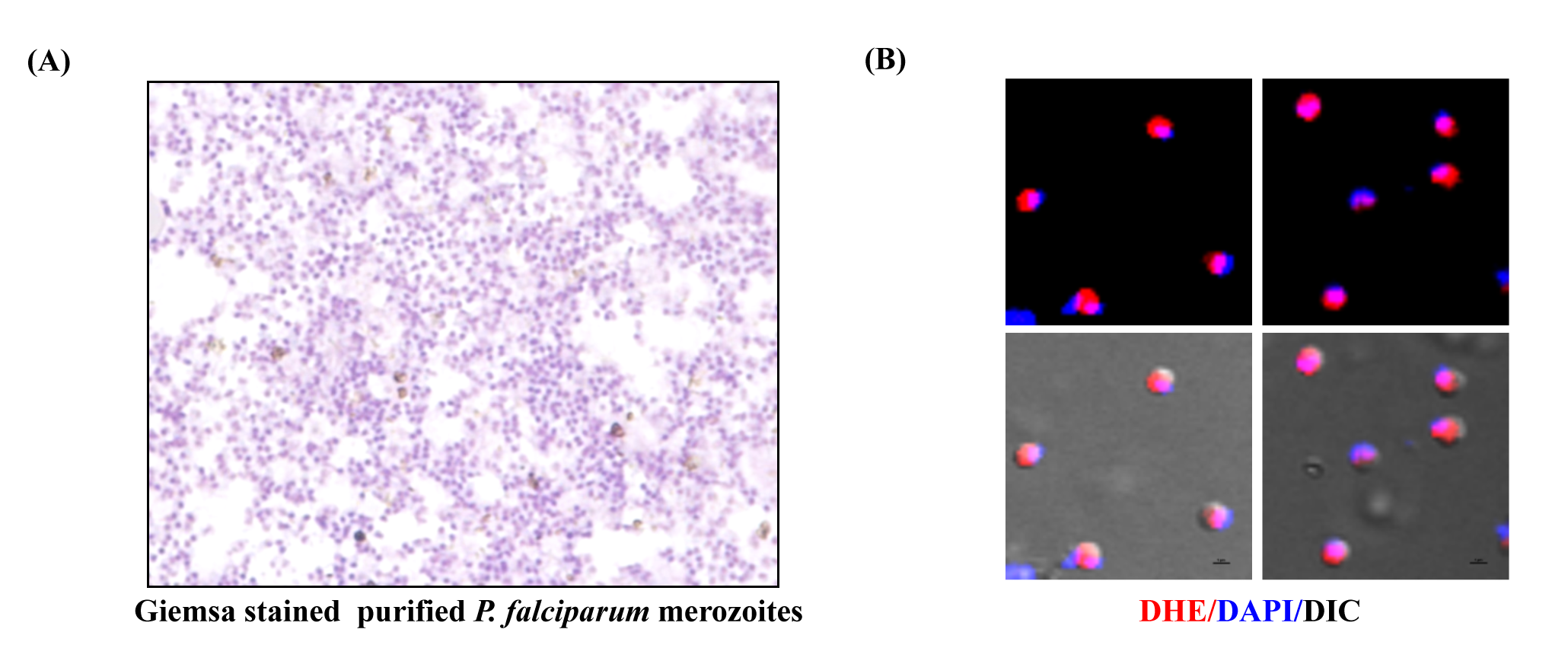

Supplement: S5 Figure — Viabilty and purity of P. falciparum merozoites. Viabilty of P. falciparum merozoites was analyzed by staining with dihydroethidine (DHE). P. falciparum merozoites were incubated with DHE (10 µg/ml) for 20 min at 37°C after purification. A) DHE-stained merozoites were further stained with nuclear staining dye DAPI and visualized using a confocal Nikon A1R microscope. B) DHE-stained merozoites were also analyzed by flow cytometry using a FACS Calibur (Becton & Dickinson, USA). 100,000 RBCs were scored per sample for fluorescence staining with DHT to determine percentage of merozoites that are viable. (TIFF) [file ppat.1004520.s005.tiff]

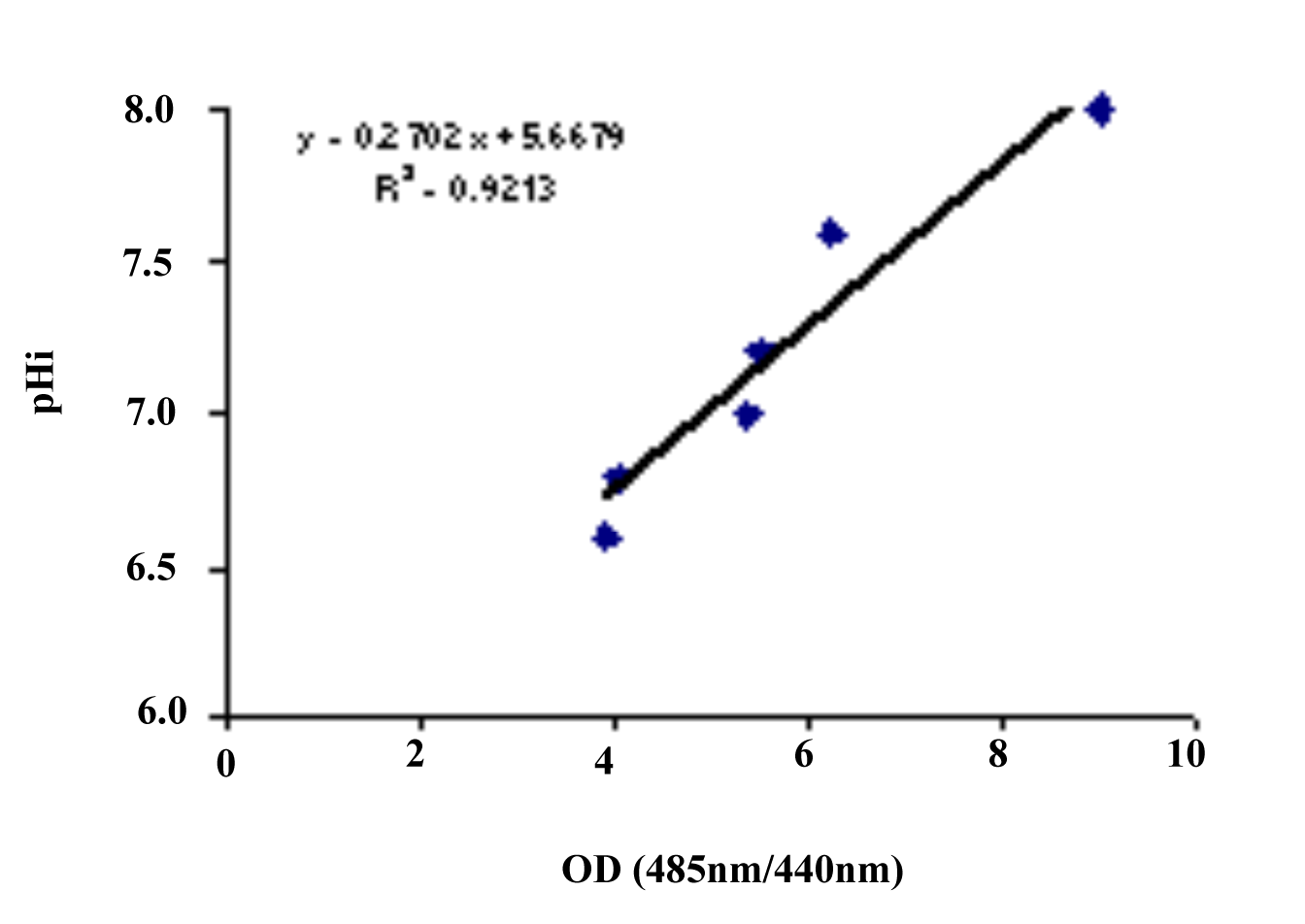

Supplement: S6 Figure — Standard curve for measurement of intracellular pH in P falciparum merozoites. P. falciparum merozoites were loaded with pH sensitive fluorescent dye BCECF-AM, resuspended in buffer at 10 different pH conditions (6.4, 6.6, 6.8, 7.0, 7.2, 7.4, 7.6, 7.8, 8.0) and treated with nigericin for 10 min at 37°C to allow extracellular and intracellular pH to equilibrate. Samples were excited at 440 nm and 492 nm and mean fluorescent intensity (MFI) was measured at 535 nm. Ratio of MFI measured at 535 nm following excitation at 492 nm and 440 nm was plotted against pH to generate a standard curve. (TIFF) [file ppat.1004520.s006.tiff]
